# Peristaltic flow of sutterby nanofluid in a stenosed artery with ciliated endothelium and wall roughness under hall and ion slip effects

**DOI:** 10.1038/s41598-026-48237-4

**Published:** 2026-05-16

**Authors:** Doaa R. Mostapha, T. M. Eldabe Nabil, W. Abbas

**Affiliations:** 1 Department of Mathematics, Faculty of Education, Ain Shams University, Roxy, Cairo, Egypt; 2 Basic and Applied Science Department, College of Engineering and Technology, Arab Academy for Science Technology, and Maritime Transport, P.O. Box 2033, Cairo, Egypt

**Keywords:** Hall implications, peristaltic stenosed artery fragments, ciliated lumen, surface roughness, and Sutterby nanofluid characteristics, Engineering, Mathematics and computing, Physics

## Abstract

In this dissertation, we examine the nonlinear peristaltic locomotion of a Sutterby nanofluid in the presence of a magnetic field from the exterior through a stenosed capillary with a ciliated endothelium lining and rough sidewalls. This study’s impetus stems from the need for more accurate hemodynamic models that can explain how magnetohydrodynamic forces and geometric irregularities alter blood flow in narrow arterial segments. Nonlinear radiant heat, viscous dissipation, and heating by Joules, Brownian diffusion, thermophoretic transport, activation energy effects, and spreading microbes are all incorporated into a comprehensive mathematical framework. Arterial roughness is modeled using a function that varies with both axial position and time, enabling representation of dynamic wall deformations. The Homotopy Perturbation Procedure is used to estimate analytical solutions for the generated equations, and qualitative compliance with experiments is provided for validation. The findings demonstrate that when the roughness amplitude rises, the critical pumping velocity falls. Furthermore, longer cilia increase hydraulic resistance and decrease axial velocity, whereas more eccentric cilia result in larger forward transport and faster flow. The originality of this integrated strategy is the simultaneous presence of time-dependent roughness and ciliated-wall mechanics for the MHD-driven artery structure. It promotes optimised performance of healthcare diagnosis and therapy, as well as constitutes a useful and accurate predictor for the evaluation of haemodynamics in stenosed arteries.

## Introduction

When compared to conventional base fluids, the addition of nanoparticles to peristaltic fluids can improve mass and heat transmission. This strategy has attracted a lot of attention. According to recent studies, nanofluids can improve thermal conductivity and change hydrodynamic behavior whenever they are undergoing peristaltic circulation. These changes could result in improved transportation techniques, temperature control, and suspended particle levels^1,2^. It turns out that tiny things like microscopic volume fraction, Brownian motion, and thermophoretic diffusion really cause changes in velocity patterns, temperature fluctuations, and concentration behavior, especially when we’re talking about lengthy wavelengths and minimal Reynolds values^3^. The study of peristaltic transport within a vertical sinusoidal wavy duct using a hybrid nanofluid composed of deionized blood and nanoparticles is examined^4^.

Sutterby fluid is a standard non-Newtonian model. It really helps us understand how shear-thinning liquids flow. Sutterby first brought up the idea back in the mid-1960s when he was looking at how fluids move in the tiny capillary tubes^5^. Sutterby’s idea is just better than simple power laws when looking at complicated stream stuff. It really gets how fluid thickness changes at different speeds^6,7^. Lately, folks have been using this framework a lot more to look at peristaltic circulation. It is a really common thing in how biological and industrial pumps work. Sutterby rheology clearly plays a role in how velocity waves, pressure changes, and shear stress shift during peristaltic motion. These neat connections to deliver medicine, make plastics, simulate how blood moves, and create tiny pumps^8^.

Arterial blood delivery hinges on peristalsis, that rhythmic contraction and relaxation. Arterial walls pulse to drive blood forward via pressure wave transmission. Wave-induced transport significantly enhances microvascular perfusion and offsets gravity’s effects in inclined arteries. Many physiological and mechanical variables influence peristaltic blood circulation. These variables are blood rheology, arterial wall compliance, the frequency and magnitude of wall contractions^9^. Computational models and experiments clarified flow configurations, velocity variations, and wall shear stresses during peristalsis. Hydrodynamic features, as peristaltic link to cardiovascular problems like hypertension, arterial stiffness, and atherosclerosis^10^. Simplifying arterial peristalsis models often hinges on assumptions about blood flow, notably low Reynolds numbers, long wavelengths, and small wave amplitudes^11^. Peristaltic circulation models are used a lot in biomedical and technological structures like medication delivery systems, artificial blood pumps, ventricular assist devices, and microfluidic platforms for controlled the transport. This ongoing investigation contributes to developing diagnostic tools and therapeutic strategies. These aimed to improve cardiovascular wellness and expanding our understanding of vascular function. Peristalsis theory is evolving, with recent work clarifying its expanded scope^2,4,6,7,12–16^.

The lumen of arteries with moderate stenosis narrows by less than half of its initial diameter. This can still have an impact on cardiovascular function. This constriction may worsen over time and raise the risk of serious cardiovascular problems. It is frequently caused by plaque buildup or early atherosclerosis^17,18^. The inner surface smoothness of arteries is important for blood transport in addition to constriction. By raising resistance and changing pressure and velocity distributions, surface roughness modifies peristaltic flow^19,20^. This impact can increase the pace of turbulent flow, promote further plaque buildup, and impair the heart’s ability to pump blood efficiently. In order to ensure smooth flow and ideal therapeutic distribution, these aspects must be taken into account. That helps in designing medical devices such as stents, catheters, and drug delivery systems. Controlling surface contacts is crucial for improving fluid movement, energy, and system longevity in industrial peristaltic pumps and microfluidic devices, in addition to biomedical applications^21,22^.

Cilia walls form structures constructed of arrays of hair-like projections located along biological passageways. The strongest evidence of cilia walls in biological passageways is found in vascular arteries. These arteries demonstrate cilia-dominated flow where flow is dictated by synchronized cilia that detect and sense flow. The ciliary propulsion plays a role in deciding upon flow velocities, fluid pressure gradients, and efficiency of transporting fluids in a peristaltic transport system. This role takes place through its interaction with traditional vessel wall contraction and subsequent relaxation. Cilia-induced flow plays an essential role in maintaining transport efficiency in cases of moderate stenosis. Moreover, cilia-induced flow minimizes shear stress, while stenosis patches regulate flow resistance. The cilia walls exhibit interaction in cases of roughness, where wall surfaces exhibit irregularities. The presence of irregularities in cilia walls significantly impacts flow, causing friction. A study employed a nanofluid model using a sophisticated model of cilia transport. It can demonstrate efficient fluid flow in transporting microorganisms due to ciliary propulsion. Consequently, this affects biomedical devices, micro lab-on-a-chip devices, and controlled drug delivery instruments^23^. New formulations on the model of nanofluidic flows due to beating cilia under the influence of the external magnetic field are investigated. This model combines the biomechanism of cilia motion, peristalsis, and texture effects, heat and mass transport. This model can use in the design of the microchannel andf microfluidic devices^24,25^.

Joule heating, Hall currents, and ion slips play an important role in conductive fluid peristalsis under specific conditions. Joule heating has considered the influence of changes in viscosity in response to changes in fluid temperature, as generated through Joule heating^26,27^. Hall currents can develop owing to effects on fluid charge under a perpendicular applied voltage^11^. Ion slips are defined as slips of ions in response to fluid flow^28,29^. They play important roles in stenosed arteries. The peristalsis has indicated its role in influencing thermal and mass transmission processes, cardiovascular and fluid dynamics problems. It has many applications, including electrokinetic drugs and smart stents/catheters^30–32^. These factors taken together inform our understanding of physiology and device design. The joint effects of Joule heating and Hall currents in peristaltic flows have a deep impact on the changes in velocity fields, pressure profiles, and thermal transport in electrically conducting fluids^33,34^. Current MHD research focuses on leveraging such interactions as an application of microfluidic pumps, advanced cooling, and peristaltic biomedical devices. The most recent work takes an immediate interest in these phenomena with theoretical insights and practical guidance in MHD systems^35,36^.

Blood and the tissues can swap fluids because the walls of the arteries are like a porous medium. Therefore, Darcy’s law doesn’t cut it when we’re talking about blood flow that pulses and artery walls^37,38^. So, scientists are using this tweaked Darcy-Forchheimer mechanism^39^. This helps to get a better handle on heart and blood vessel problems. It can improve medical devices such as stents and grafts^40–42^. These interactions really change how pressure gets spread around, how much the flow resists, and how things get moved^43–45^.

This paper deals with peristaltic flow in stenosed and rough- wall arteries filled with Sutterby nanofluid that are not treated simultaneously in previous work. The present model of cilia- wall, chemical reactions, motilegyrotactic microorganisms is investigated. This model further considers Hall currents, Joule heating, and ion-slip consequences with MHD effects. Non-linear contributions from the altered Darcy-Forchheimer model are incorporated with stenosed and porous arterial walls. Soret and Dufour effects, in conjunction with Arrhenius activation energy, are invoked for investigation. This study also includes chemical reaction phenomena whereas viscous dissipation accounts for energy dissipation in the system. This approach represents a novel advancement over previous work by combining peristaltic dynamics, Sutterby nanofluid, MHD, stenosed and rough ciliated wall, chemical reactions, and microorganism structures. By utilizing a very low Reynolds ratio as well as an extended wavelength, partial differential equations that regulate may transform into simpler forms of differential equations. Homotopy perturbation methodology introduced a powerful tool in that it produces knowledge concerning perturbation procedures. The results are presented in a graphical form concerning temperature, concentration, velocity, pressure rise, frictional forces, as well as microscopic organism patterns. Skin friction, local concentration of motile microorganisms, as well as non-dimensional values such as Nusselt and Sherwood components, are additionally considered in this research study.

This integrated framework provides a more realistic representation of blood flow in diseased arteries and helps to better understand hemodynamic behavior relevant to biomedical diagnostics and therapeutic applications, such as targeted drug delivery systems that utilize peristaltic motion and enhanced nanoparticle transport. Design and optimization of microfluidic devices for controlled fluid and particle flow. Development of advanced stents and vascular grafts that account for wall roughness and fluid dynamics. Diagnostics and monitoring of cardiovascular diseases, particularly stenosis and atherosclerosis. Artificial organ and tissue engineering, simulating realistic microcirculation conditions. Biomedical heat and mass transfer systems improve the cooling or heating of biological fluids. Lab-on-a-chip platforms for studying microorganism behavior and bioconvection effects. Optimization of biosensors that rely on ion transport and electrokinetic phenomena. Modeling and simulation of blood flow dynamics in complex geometries for clinical planning. Thus, this study provides a practical insight into blood flow dynamics in arteries affected by stenosis and surface roughness due to atherosclerotic plaques, which are composed of fatty deposits, cholesterol, cellular debris, calcium, and fibrin accumulating on the arterial walls. Thus, due to this effect of cilia on flow field patterns, mixing enhancement, and alteration of stress fields, another complexity has come into the problem of modeling blood flow in a rough artery surface due to the presence of cilia structures on these surfaces. The realistic depiction of physiological processes occurring on a microscopic level in this phenomenon of interplay between cilia structures and rough surfaces of arteries indicates that these kinds of biological structures can reduce or enhance further problems of disruptions in blood flow due to the presence of blocks of plaque. Such a phenomenon also serves as a realistic representation of how concepts and results generated in this study can be practically implemented.

To better understand the complex interactions among peristaltic motion, ciliated endothelium, wall roughness, and magnetohydrodynamic effects, this study addresses several key research questions: How does the peristaltic motion of the arterial wall influence the velocity distribution and flow patterns of Sutterby nanofluid in stenosed arteries? What is the effect of time-dependent wall roughness on hydraulic resistance and pressure distribution within the arteries? How do cilia motion and wall roughness interact to modify heat transfer and thermal transport in the nanofluid? What role do Hall and ion-slip effects play in the behavior of electrically conducting nanofluids in narrow arterial segments?

By applying a dynamic method to calculate surface roughness and by adding time as an explicit variable in the surface equation, this study introduces another key improvement. In this model, the equation depends on both spatial position and time, enabling it to represent arterial wall geometry rather than only spatial variation, as in classical approaches. This shows how ciliary motion interacts with uneven arterial surfaces in a nontrivial manner. Because some properties of arterial surfaces vary over time, it is possible to model a range of physiological processes with better accuracy and precision, but this time dependence also makes the analysis more complex. Treatments that account for time-varying features of blood flow may improve health outcomes and open up new directions for medical engineering and therapy development.

## The organizational structure of the issue

The present study investigates the peristaltic flow of Sutterby nanofluid containing gyrotactic microorganisms that are motile through an artery featuring surface roughness and ciliated walls. The arterial wall shape is sinusoidal, with a region that undergoes antagonistic stenosis to allow realistic physiological representation. The fluid entering the artery has to pass through a porous medium modeled via a modified Darcy-Forchheimer model. Besides, the system under consideration is subjected to a strong uniform magnetic field ${B_0}$. The presence of cilia on the arterial walls affects the near-wall flow phenomena considerably, enhances the mixing phenomenon, and thereby interacts with both the roughness and stenotic features in a complicated manner, and the velocity distribution, pressure gradients, and nanoparticle transport throughout the artery. The combined impact of ion-slip, dissipation due to viscosity, Joule heating, frictional heating, and Hall currents is studied. The connection between the chemical and heat radiation is analyzed by considering the Soret and Dufour diffusion. The nanoparticles’ concentration is controlled by using activation energy along with Buongiorno’s method and the modified Arrhenius expression.

**Fig. 1:**
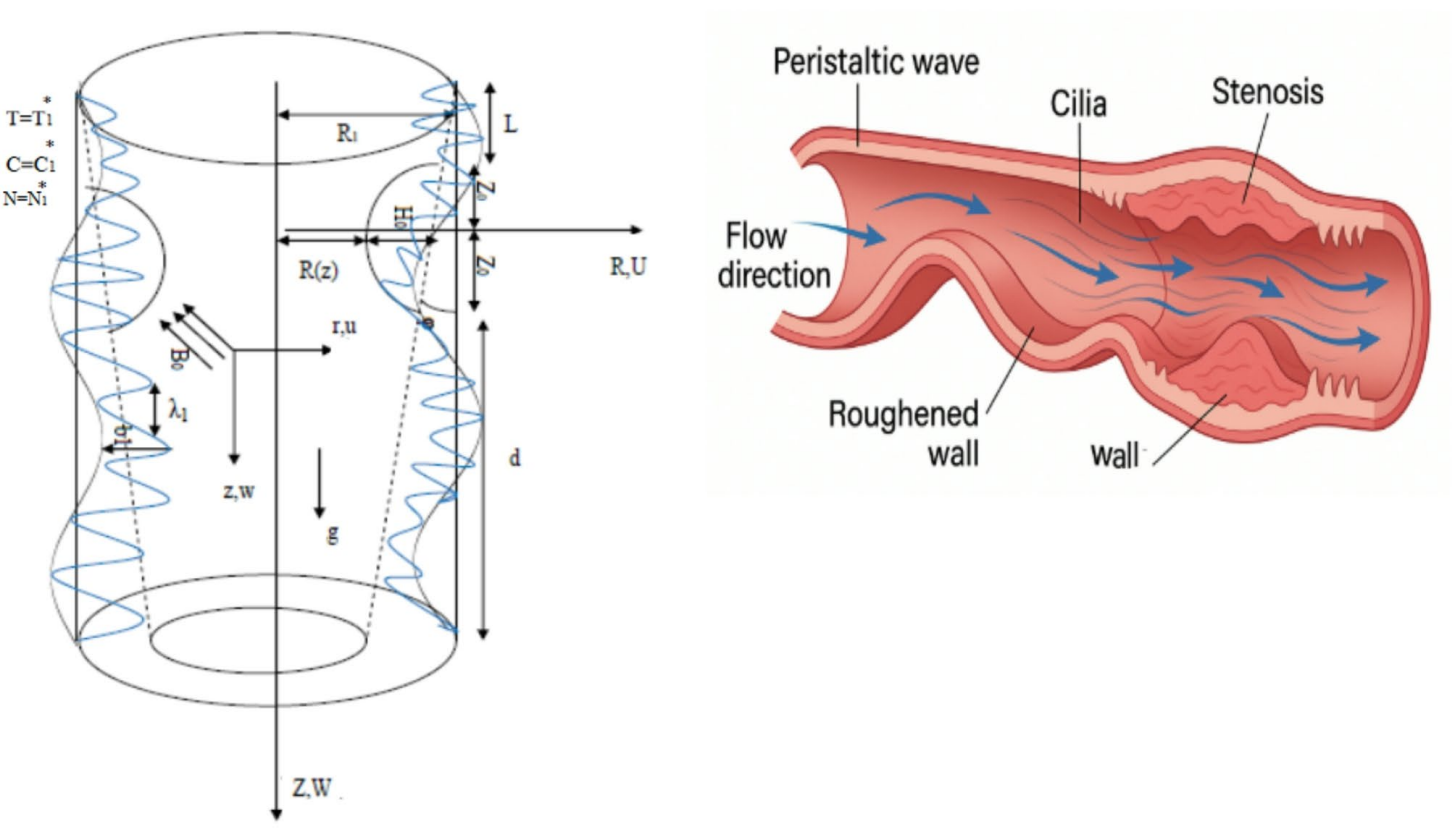
Modeling of the system’s intrinsic structural features alongside its practical application in real-world scenarios.

Meanwhile, the study includes an electric field. The procedure of cylindrical coordinates (*R*, $\theta$, *Z*) is impacted with the tube axis in the path of the *Z*-axis. The Hall principle occurs in the $\theta$-path. However, there is no impact of microorganisms, concentration fields, temperature distribution, and velocity structure in the $\theta$-path. The velocity slip condition is utilized. Moreover, as described in Fig. 1, the artery is subjected to thermal impacts, concentration, and microorganism denoted by $T_{1}^{{{*}}}$, $C_{1}^{{{*}}}$, $N_{1}^{{{*}}}$, respectively. The Reynolds values are taken to be very tiny. So, the induced magnetic field has a little impact compared to the applied exterior magnetic field. As a result, the implication of the induced magnetic field can be disregarded.

The alteration of radius $R\left( z \right)$ is^18^:

1 $$R\left( z \right)=\left\{ {\begin{array}{*{20}{l}} {{R_1} - \delta {m_1}\left( {Z+L+d} \right)}&{~~~~ - {{L}} \leqslant {{Z}}< - {{{Z}}_0},} \\ {{R_1} - \delta {m_1}\left( {Z+L+d} \right) - \frac{{{H_0}}}{2}\left[ {1+{{cos}}\frac{{\pi Z}}{{{Z_0}}}} \right]}&{~~~~ - {{{Z}}_0} \leqslant {{Z}} \leqslant {{{Z}}_0},} \\ {{R_1} - \delta {m_1}\left( {Z+L+d} \right)}&{~~~~~~~{{{Z}}_0}<{{Z}} \leqslant {{d}},} \end{array}} \right.$$

$${{where}},~\delta =\frac{{{R_1}}}{\lambda },{{~}}{H_0}=h{{cos}}\phi {{~and~}}{m_1}={{tan}}\phi .$$

${H_0}$ to *r* is less than 1. The artery length is bounded by $L+d$^18^. In $\phi <0$, the convergence happens, the divergence portion is noted at $\phi >0$, and the untapered vasculature is detected at $\phi =0$^22,26^.

The Sutterby rheological model, which forms the core theoretical foundation of the study, is used to characterize the fluid’s non-Newtonian nature. As a result, the appropriate constitutive relation that controls the flow behavior is expressed as follows^5^:

2 $$\underline{\underline{\sigma }} ^{*} = - PI + \underline{\underline{\tau }} ,$$

3 $$\underline{\underline{\tau }} = \frac{{\mu _{f} }}{2}\left[ {\frac{{\sinh ^{{ - 1}} \left( {n_{1} ~\dot{\gamma }} \right)}}{{n_{1} ~\dot{\gamma }}}} \right]^{m} A_{1} ,$$

where ${{\dot {\boldsymbol{\upgamma}}}}=\sqrt {\frac{{{\boldsymbol{\uppi}}}}{2}}$, ${{\boldsymbol{\uppi}}}={{{t}}_{{r}}}{{A}}_{1}^{2}$. When the incompressibility condition is imposed, the isotropic component of the stress tensor is represented by the term $-{{PI}}$ and

4 $${A_1}=\nabla \underset{\raise0.3em\hbox{$\smash{\scriptscriptstyle-}$}}{V} +\nabla {\underset{\raise0.3em\hbox{$\smash{\scriptscriptstyle-}$}}{V} ^T}.$$

For different values of the material parameter *m*, the fluid response changes in a physically meaningful way. When $m=0$, the constitutive relation reduces to that of a classical Newtonian fluid. For $m=1$, the model represents a weakly non-Newtonian fluid, where the shear stress remains nearly proportional to the shear rate, with only a slight deviation introduced through the term ${\sinh ^{ - 1}}\left( {{n_1}~\dot {\gamma }} \right)$. For $m>0$, the fluid exhibits shear-thickening characteristics, meaning that the shear stress grows more rapidly than the shear rate, reflecting an apparent increase in viscosity at higher deformation rates. In contrast, for $m<0$, the fluid displays shear-thinning behavior, where the shear stress rises more slowly as the shear rate increases, indicating a reduction in effective viscosity and a tendency toward pseudoplastic flow.

To avoid the mathematical complexities in the equations, the inverse sine function in an approximated form may also follow the Taylor series value. Here also the condition is applicable as $m~ \ll 1$. i.e., the initial two terms in the Taylor series may be considered while the other terms may be neglected since they involve nonlinear values. Thus, the non-Newtonian effect as per the Sutterby theory can be obtained along with the simplicity in the equations. Thus, Eq. (3) may be simplified as:

5 $$\underline{\underline{\tau }} = \frac{{\mu _{f} }}{2}\left[ {1 - \frac{{mn_{1}^{2} ~\dot{\gamma }^{2} }}{6}~} \right]A_{1} ,$$

In view of the incompressibility assumption, the mass conservation requirement simplifies, leading to a modified form of the continuity equation. 

6 $$\nabla .\underset{\raise0.3em\hbox{$\smash{\scriptscriptstyle-}$}}{V} =0.$$

The fundamental neutralization that controls fluid flow is described mathematically as follows^22^:

7 $$\rho _{f} \left( {\frac{{\partial \underset{\raise0.3em\hbox{$\smash{\scriptscriptstyle-}$}}{V} }}{{\partial t}} + \left( {\underset{\raise0.3em\hbox{$\smash{\scriptscriptstyle-}$}}{V} .\nabla } \right)\underset{\raise0.3em\hbox{$\smash{\scriptscriptstyle-}$}}{V} } \right) = - \nabla P + \nabla .\underline{\underline{\tau }} + \underset{\raise0.3em\hbox{$\smash{\scriptscriptstyle-}$}}{J} \wedge \underset{\raise0.3em\hbox{$\smash{\scriptscriptstyle-}$}}{B} + \underset{\raise0.3em\hbox{$\smash{\scriptscriptstyle-}$}}{R} _{D} - \frac{{\rho _{f} c_{b} }}{{\sqrt {k^{*} } }}\underset{\raise0.3em\hbox{$\smash{\scriptscriptstyle-}$}}{V} \left| {\underset{\raise0.3em\hbox{$\smash{\scriptscriptstyle-}$}}{V} } \right| + \rho _{f} \underset{\raise0.3em\hbox{$\smash{\scriptscriptstyle-}$}}{g} ,$$

where

8 $${\underset{\raise0.3em\hbox{$\smash{\scriptscriptstyle-}$}}{R} _D}= - \frac{{{\epsilon ^*}{\mu _f}}}{{2{k^*}}}\left( {1 - \frac{{mn_{1}^{2}~{{\dot {\gamma }}^2}}}{6}} \right)\underset{\raise0.3em\hbox{$\smash{\scriptscriptstyle-}$}}{V} ,$$

$$\underset{\raise0.3em\hbox{$\smash{\scriptscriptstyle-}$}}{g} =\left( {0,0, - g} \right)$$, $$\underset{\raise0.3em\hbox{$\smash{\scriptscriptstyle-}$}}{J} \wedge \underset{\raise0.3em\hbox{$\smash{\scriptscriptstyle-}$}}{B}$$ implies the presence of the Lorentz force, and $$\underset{\raise0.3em\hbox{$\smash{\scriptscriptstyle-}$}}{B} =\left( {0,~{B_0},0} \right)$$ flows along a route that is orthogonal.

The following is the generalized Ohm’s concept that describes circulation in the light of both Hall and ion-slip variations[27 and 28]:

9 $$\underset{\raise0.3em\hbox{$\smash{\scriptscriptstyle-}$}}{J} = \sigma _{{hnf}} \left( {\widehat{{\underset{\raise0.3em\hbox{$\smash{\scriptscriptstyle-}$}}{E} }} + ~\underset{\raise0.3em\hbox{$\smash{\scriptscriptstyle-}$}}{V} \wedge \underset{\raise0.3em\hbox{$\smash{\scriptscriptstyle-}$}}{B} } \right) + \frac{{\varpi _{e} ~\tau _{e} }}{{B_{0} }}\left( {\underset{\raise0.3em\hbox{$\smash{\scriptscriptstyle-}$}}{J} \wedge \underset{\raise0.3em\hbox{$\smash{\scriptscriptstyle-}$}}{B} } \right) + \frac{{\varpi _{e} ~\tau _{e} \beta _{i} }}{{B_{0}^{2} }}\left( {\underset{\raise0.3em\hbox{$\smash{\scriptscriptstyle-}$}}{J} \wedge \underset{\raise0.3em\hbox{$\smash{\scriptscriptstyle-}$}}{B} } \right) \wedge \underset{\raise0.3em\hbox{$\smash{\scriptscriptstyle-}$}}{B} ,$$

The third term represents the influence of electron drag on ions, commonly referred to as the ion-slip effect. The second term corresponds to the Hall effect, which arises from the relative motion between electrons and ions when they decouple under an electromagnetic field. This decoupling generates a drift between charged particles and neutral species, particularly when $${\varpi _e}~{\tau _e}\sim O\left( 1 \right)$$along with $${\varpi _i}~{\tau _i} \ll 1$$. In this analysis, no external electric field is considered, meaning the polarization of the ionized fluid is neglected, and it is assumed that $$\widehat {{{{\underset{\raise0.3em\hbox{$\smash{\scriptscriptstyle-}$}}{E} }}}}=0$$, so both applied and induced electric fields have negligible effects on the flow^28^.

The overall density of the nanofluid is defined by the combination of the densities of its constituent fluid and suspended nanoparticles as follows^2^:

10 $$\rho = - {\rho _f}{T_e}\left( {1 - C_{1}^{{{*}}}} \right)\left( {T - T_{1}^{{{*}}}} \right)+\left( {{\rho _p} - {\rho _f}} \right){C_e}\left( {C - C_{1}^{{{*}}}} \right)+\left( {N - N_{1}^{{{*}}}} \right)\vartheta \left( {{\rho _m} - {\rho _f}} \right),$$

The thermal transport equation, accounting for both nonlinear radiation and Dufour-induced heat flux, is given by^37^:

11 $$\begin{aligned} {(\rho c)_f}\left( {\frac{{\partial T}}{{\partial t}}+\underset{\raise0.3em\hbox{$\smash{\scriptscriptstyle-}$}}{V} .\nabla T} \right) & =K{\nabla ^2}T - \nabla .{q_r}+\frac{1}{{{\sigma _{hnf}}}}\underset{\raise0.3em\hbox{$\smash{\scriptscriptstyle-}$}}{J} .\underset{\raise0.3em\hbox{$\smash{\scriptscriptstyle-}$}}{J} +\frac{{{\epsilon ^*}{\mu _0}}}{{{k^*}}}\left( {1 - \frac{{mn_{1}^{2}~{{\dot {\gamma }}^2}}}{6}} \right)\left( {\underset{\raise0.3em\hbox{$\smash{\scriptscriptstyle-}$}}{V} ~.\underset{\raise0.3em\hbox{$\smash{\scriptscriptstyle-}$}}{V} ~} \right)\left| {\underset{\raise0.3em\hbox{$\smash{\scriptscriptstyle-}$}}{V} } \right|~~ \\ ~ & \;\;+~{(\rho c)_p}\left( {{D_B}\nabla C.\nabla T+\frac{{{D_T}}}{{T_{1}^{{{*}}}}}\nabla T.\nabla T} \right)+\frac{{D{K_T}}}{{{C_s}}}{\nabla ^2}C+{{\boldsymbol{\Phi}}}+{Q_0}, \\ \end{aligned}$$

12 $${{\boldsymbol{\Phi}}}={\tau _{RR}}\frac{{\partial U}}{{\partial R}}+{\tau _{RZ}}\left( {\frac{{\partial U}}{{\partial Z}}+\frac{{\partial W}}{{\partial R}}} \right)+{\tau _{ZZ}}\frac{{\partial W}}{{\partial Z}},$$

$${{where~}}k=\frac{{{K}}}{{{{(\rho c)}_f}}},{{~}}\underset{\raise0.3em\hbox{$\smash{\scriptscriptstyle-}$}}{V} =\left( {{{U}},0,{{W}}} \right){{~and~}}\left| {\underset{\raise0.3em\hbox{$\smash{\scriptscriptstyle-}$}}{V} } \right|=\sqrt {{U^2}+{W^2}} .$$

Following the Rosseland approach^43^, the radiative heat flux in a nonlinear medium is expressed in the form below:

13 $${q_r}= - \frac{{4{\sigma _0}}}{{3{K_0}}}\frac{{\partial {T^4}}}{{\partial R}},$$

When temperature fluctuations are minimal, the term ${{{T}}^4}$ can be roughly described as linearly dependent on temperature. The nonlinear radiant temperature variation may be made simpler by extending the Taylor distribution around the median temperature ${T_m}$ and ignoring higher-order terms.

14 $${q_r}= - \frac{{16{\sigma _0}}}{{3{K_0}}}{T_m}\frac{{\partial T}}{{\partial R}}.$$

The following formulation describes species concentration considering chemical interactions and energy-dependent activation^1^:

15 $$\frac{{\partial C}}{{\partial t}}+\underset{\raise0.3em\hbox{$\smash{\scriptscriptstyle-}$}}{V} .\nabla C={D_B}{\nabla ^2}C+\frac{{{D_T}{K_T}}}{{T_{1}^{*}}}{\nabla ^2}T - k_{r}^{2}\left( {C - C_{1}^{{{*}}}} \right){\left( {\frac{T}{{T_{1}^{{{*}}}}}} \right)^n}{{exp}}\left( {\frac{{ - {E_a}}}{{{\omega ^*}T}}} \right),$$

where ($- 1<n<1$). At the ending of the Eq. (15) comes an expression known as the “Arrhenius principle,” which summarizes all of the impacts of chemical reaction and activation energy within the structure of a nanofluid.

The motion of microbes is dictated by the next formula^43^:

16 $$\frac{{\partial N}}{{\partial t}}+\underset{\raise0.3em\hbox{$\smash{\scriptscriptstyle-}$}}{V} .\nabla N+\frac{{b{\omega _c}}}{{C_{1}^{{{*}}}}}\nabla .\left( {N\nabla C} \right)={D_{m0}}{\nabla ^2}N,$$

Cilia are microscopic, hair-like structures distributed along the inner walls of many biological conduits. Their coordinated beating generates localized fluid motion, enhances mixing, and assists transport mechanisms in physiological systems. In arterial and microfluidic environments, ciliated walls can effectively regulate flow behavior, interact with peristaltic waves, and influence shear stress, heat transfer, and mass diffusion, especially when surface roughness and non-Newtonian fluid characteristics are present. For a ciliated wall, the kinematics of cilia motion are described through the spatial position of the cilia tips. Consequently, the cilia tips’ displaced horizontally may be written as^46^:

17 $$\zeta \left( {{{z}};{{t}}} \right)={\xi _0}+\alpha \xi {R_1}{{sin}}\frac{{2\pi }}{\lambda }\left( {Z - \frac{{kt}}{{{R_1}}}} \right)~,$$

For instance, studies report that the paths traced by cilia tips are usually elliptical in flows driven by ciliary motion, which are often described as peristaltic flow. A particle’s velocity profile and transport depend on its interaction with the fluid, which changes with the radial position of the cilium tip. The motion at the cilium tip is necessary for simulating flow in microchannels and in biological settings, including arteries narrowed by stenosis, and it can be written as follows^46^:

18 $$\eta \left( {{{Z}};{{t}}} \right)={R_1}+\xi {R_1}{{cos}}\frac{{2\pi }}{\lambda }\left( {Z - \frac{{kt}}{{{R_1}}}} \right)~,$$

For peristaltic circulation, the geometry of the rough ciliated wall can be formulated as^10,19,20^, and^46^:

19 $${H_1}\left( {{{z}};{{t}}} \right)=a~{{cos}}\frac{{2\pi }}{\lambda }\left( {Z - \frac{{kt}}{{{R_1}}}} \right) - {b_1}{{co}}{{{s}}^4}\left( {\frac{{\pi ~Z}}{{{\lambda _1}}}} \right)+{{\boldsymbol{\upeta}}}\left( {{{Z}},{{t}}} \right)$$

The novel aspect of this paper is the existence of wall roughness (a function of both space and time ) with cilia streams.

The preset velocities on the boundary considered the no-slip condition. The preset temperature and concentration boundary condition is to consider the impacts of Soret, Dufour, radiation, viscous, and Joule heating. In the microorganisms problem, the boundary condition attempts to account for the diffusive motion, gyrotaxis, and motility of motile bacterial cells on the surface. The condition for a rough artery wall is also a function of time and space. The presence of cilia is also found. In contrast to earlier models that merely took into account smooth walls or static roughness, this combination incorporates dynamic interactions between the wall, cilia, and fluid.

The conditions under the circumstances can be expressed as

20 $$U=\frac{{\partial {H_1}}}{{\partial t}},~W=\frac{{\partial \zeta }}{{\partial t}} - \gamma {\tau _{RZ}},~T=T_{1}^{{{*}}},~C=C_{1}^{{{*}}},~N=N_{1}^{{{*}}}~~~at~~~~R={R_2}=R\left( z \right)+{H_1},$$

21 $$U=0,~~~\frac{{\partial W}}{{\partial R}}=\frac{{\partial T}}{{\partial R}}=\frac{{\partial C}}{{\partial R}}=~\frac{{\partial N}}{{\partial R}}=0~at~~~R=0,~~~~$$

To ensure clarity in the problem formulation, the boundary conditions are specified in detail to reflect the physical and physiological characteristics of the system. For the ciliated wall, the kinematics of cilia motion are described by the spatial position of the cilia tips, which move in elliptical trajectories, generating localized fluid motion and enhancing mixing. The wall roughness is modeled as a function of both space and time, allowing representation of dynamic deformations in stenosed arteries. The velocity boundary conditions follow the classical no-slip assumption at the wall, while the temperature and concentration boundary conditions account for effects such as Soret and Dufour diffusion, radiation, viscous dissipation, and Joule heating. In addition, for motile microorganisms, the boundary conditions include diffusive motion, gyrotaxis, and bacterial motility along the surface. At the artery centerline, symmetry conditions are applied, with zero velocity and zero gradients for temperature, concentration, and microorganism density. These boundary conditions ensure that the model realistically captures the coupled interactions between peristaltic motion, cilia, wall roughness, fluid transport, thermal and concentration fields, and microorganisms, providing a comprehensive framework for analyzing flow in stenosed ciliated arteries.

The rate of fluid volume transport in a fixed geometry is expressed by the following relation^10^:

22 $${{\Xi }} = 2\pi \mathop \smallint \limits_{0}^{{R_{2} }} WRdR,$$

$${R_2}={R_2}\left( {Z,t} \right).$$

According to the position of Z, the averaged over time circulating rate $\hat {Q}$ during timeframe $\hat {\tau }=\frac{{\lambda {R_1}}}{k}$ is captured as^10^:

23 $$\hat{Q} = \frac{1}{{\hat{\tau }}}\mathop \smallint \limits_{0}^{{\hat{\tau }}} \Xi d\hat{\tau }.$$

The oscillatory input in the lab framing $\left( {R,{{~~}}0,~~Z} \right)$ may be interpreted as an uninterrupted stream in the wave framing $\left( {r,0,z} \right)$, maintaining that pressure remains unchanged at the tube borders. These coordinates’ connection is stated as^10^:

24 $$u=U,~~~w=W - \frac{k}{{{R_1}}},~~~r=R,~~~~{{and}}~~~~z=Z - \frac{k}{{{R_1}}}t.$$

The partial differential equations that determine the direction of fluid motion can be reduced into a more understandable form by using the idea of prolonged wavelengths and very tiny Reynolds amounts^10^. By reducing the pertinent elements, these equations can subsequently be represented in dimensionless form. In particular, the length scaling ${R_1}$ and $\lambda$ besides the mass component M, result in non-dimensional formulas. Additional pertinent non-dimensional quantities in the scheme are^26^:

25 $$\begin{aligned} \bar {r} & =\frac{r}{{{R_1}}},~~~~\bar {z}=\frac{z}{\lambda },~~~~\bar {u}=\frac{{u{R_1}}}{{k\delta }},~~~\bar {w}=\frac{{w{R_1}}}{k},~~~~\overline {{{H_1}}} =\frac{{{H_1}}}{{{R_1}}},~~\delta =\frac{{{R_1}}}{\lambda },~~\bar {S}=\frac{{SR_{1}^{2}}}{{\mu k}},~~~~ \\ \overline {{{z_0}}} & =\frac{{{z_0}}}{\lambda },~~\bar {L}=\frac{L}{\lambda },~~~\bar {h}=\frac{h}{{{R_1}}},~~~~\bar {d}=\frac{d}{\lambda },~~~~\overline {{R\left( z \right)}} =\frac{{R\left( z \right)}}{{{R_1}}},~~~~\bar {P}=\frac{{PR_{1}^{3}}}{{\mu \lambda k}},{{~}}~\overline {{{k^*}}} =\frac{{{k^*}}}{{R_{1}^{2}}},~~~~ \\ \bar {T} & =\frac{{T - T_{1}^{{{*}}}}}{{\beta {R_1}}},~~~\bar {C}=\frac{{C - C_{1}^{{{*}}}}}{{C_{1}^{{{*}}}}},~~~\bar {N}=\frac{{N - N_{1}^{{{*}}}}}{{N_{1}^{{{*}}}}},~\bar {t}=\frac{k}{{{R_1}\lambda }}t,~~\overline {{{\alpha ^*}}} =\frac{{{\alpha ^*}}}{\delta }~~{{and}}~~~~\bar {Q}=\frac{Q}{{2\pi {R_1}k}}. \\ \end{aligned}$$

The remaining dimensionless quantities are outlined below:

$${R_N}=\frac{{R_{1}^{3}{C_e}\left( {C_{0}^{{{*}}} - C_{1}^{{{*}}}} \right)g\left( {{\rho _p} - {\rho _f}} \right)}}{{{\mu _0}k}},{G_r}=\frac{{{\rho _f}{T_e}\left( {1 - C_{1}^{{{*}}}} \right)\left( {\beta {R_1}} \right)R_{1}^{3}g}}{{{\mu _0}k}},$$

$${R_b}=\frac{{R_{1}^{3}g\left( {N_{0}^{{{*}}} - N_{1}^{{{*}}}} \right)\left( {{\rho _m} - {\rho _f}} \right)\vartheta }}{{{\mu _0}k}},{D_{Ma}}=\frac{{{k^*}}}{{{\epsilon ^*}R_{1}^{2}}},{F_r}=\frac{{{c_b}{R_1}k}}{{\nu \sqrt {{k^*}} }},{R_e}=\frac{k}{\nu },{{{S}}_{Tb}}=\frac{{{{n~}}{{{m}}^2}~{k^2}}}{{6~R_{1}^{4}}},{\varepsilon _1}=a/{R_1},$$

$${H^2}=\frac{{\sigma B_{0}^{2}R_{1}^{2}}}{{{\mu _0}}},{\alpha _e}=1+{\beta _e}{\beta _i},{P_r}=\frac{{{\mu _0}}}{{\rho k}},{S_c}=\frac{k}{D},{N_b}=\frac{{{\tau _c}{D_B}\left( {C_{0}^{{{*}}} - C_{1}^{{{*}}}} \right)}}{k},{N_t}=\frac{{{\tau _c}{D_T}\left( {\beta {R_1}} \right)}}{{kT_{1}^{{{*}}}}},$$

$${E_c}=\frac{{{k^2}}}{{R_{1}^{3}{c_f}\beta }},{B_r}={P_r}{E_c},{R_n}=\frac{{16{\sigma _0}T_{m}^{3}}}{{3K{K_0}}},{D_u}=\frac{{D{K_T}\left( {C_{0}^{{{*}}} - C_{1}^{{{*}}}} \right)}}{{{\mu _0}{C_s}\beta {R_1}{c_f}}},\hat {\alpha }=\frac{{k_{r}^{2}R_{1}^{2}}}{k},{\beta _t}=\frac{{\beta {R_1}}}{{T_{1}^{{{*}}}}},{{\rm E}_{\boldsymbol{a}}}=\frac{{{E_a}}}{{{\omega ^*}T_{1}^{{{*}}}}},$$

$${P_e}=\frac{{b{\omega _c}}}{{{D_{m0}}}},{\gamma ^{{*}}}= - \frac{{\gamma {\mu _0}}}{{{R_1}}},{Q^*}=\frac{{{Q_0}R_{1}^{2}}}{{K\left( {\beta {R_1}} \right)}},{\varepsilon _2}={b_1}/{R_1},{{and~}}{{{\boldsymbol{\uplambda}}}^*}=\frac{{{\boldsymbol{\uplambda}}}}{{{{{\boldsymbol{\uplambda}}}_1}}}.$$

Overbar notation is used to represent all dimensionless variables. This notation is left out for ease of use and clarity in the analysis that follows. Furthermore, the radial velocity component *u* is usually much less in magnitude than the axial velocity component *w*, therefore it can be considered unimportant in relative terms^18^. Owing to the micro-scale action of the cilia and the presence of surface roughness along the arterial wall, the axial transport induced by peristaltic waves is significantly more dominant than the radial motion of the fluid. The coordinated beating of cilia primarily enhances fluid propulsion in the axial direction, while roughness elements introduce localized radial disturbances that remain comparatively weak. Consequently, variations of the axial velocity along the longitudinal direction are much smaller than those across the radial direction, leading to the physically reasonable assumptions $u<<w$ and $\frac{{\partial w}}{{\partial z}}<<\frac{{\partial w}}{{\partial r}}$. In addition, the long-wavelength nature of peristaltic waves generated by ciliary activity, together with the slow flow regime typical of physiological conditions, justifies considering a very small wavelength ratio $\delta <<1$. This small parameter naturally arises from the interaction between cilia-induced wall motion and time-dependent surface roughness. Therefore, a perturbation framework based on the smallness of $\delta$ is adopted to simplify the governing Eqs. (2–23). and obtain approximate analytical solutions. This asymptotic approach captures the essential physics of cilia–roughness coupling while retaining the dominant features of peristaltic transport. As a result, the governing momentum, energy, concentration, and microorganism transport equations reduce to simplified forms after applying these physically justified approximations as follows:

The momentum balance along the radial direction reduces to the following form:

26 $$\frac{{\partial P}}{{\partial r}}=0$$,

Despite the presence of cilia-induced wall motion and surface roughness effects, Eq. (26) reveals that the pressure gradient remains independent of the radial coordinate, and therefore, the pressure *P* varies only along the axial path $\left( z \right)$.

As a result of the adopted approximations, the momentum equation along the axial (*z*) direction takes the reduced form given below:

27 $$\begin{aligned} \frac{{\partial P}}{{\partial z}} & =\frac{1}{r}\frac{\partial }{{\partial r}}\left\{ {\left( {1 - {S_{Tb}}{{\left( {\frac{{\partial w}}{{\partial r}}} \right)}^2}} \right)\left( {r\frac{{\partial w}}{{\partial r}}} \right)} \right\} - {F_r}{(w+1)^2}+{G_r}T - {R_N}C - {R_b}N \\ & \;\;~ - ~\left[ {\frac{{~{\alpha _e}{H^2}}}{{{{~}}\alpha _{e}^{2}+\beta _{e}^{2}}}+\frac{1}{{{D_{Ma}}}}\left( {1 - {S_{Tb}}{{\left( {\frac{{\partial w}}{{\partial r}}} \right)}^2}} \right)} \right]\left( {w+1} \right), \\ \end{aligned}$$

The resulting form of the energy balance equation can be written as:

28 $$\begin{aligned} \left( {1+{R_n}} \right)\frac{1}{r}\frac{\partial }{{\partial r}}\left( {r\frac{{\partial T}}{{\partial r}}} \right) & +{D_u}{P_r}\frac{1}{r}\frac{\partial }{{\partial r}}\left( {r\frac{{\partial C}}{{\partial r}}} \right)+{B_r}\left( {\frac{{~{H^2}}}{{{{~}}\alpha _{e}^{2}+\beta _{e}^{2}}}+\frac{1}{{{D_{Ma}}}}\left( {1 - {S_{Tb}}{{\left( {\frac{{\partial w}}{{\partial r}}} \right)}^2}} \right)} \right){(w+1)^2} \\ & +{B_r}\left( {1 - {S_{Tb}}{{\left( {\frac{{\partial w}}{{\partial r}}} \right)}^2}} \right){(\frac{{\partial w}}{{\partial r}})^2}+{N_b}\frac{{\partial C}}{{\partial r}}\frac{{\partial T}}{{\partial r}}+{N_t}{(\frac{{\partial T}}{{\partial r}})^2}+{{{Q}}^*}=0. \\ \end{aligned}$$

The evolution of species concentration is given by:

29 $$\frac{1}{r}\frac{\partial }{{\partial r}}\left( {r\frac{{\partial C}}{{\partial r}}} \right)+\frac{{{N_t}}}{{{N_b}r}}\frac{\partial }{{\partial r}}\left( {r\frac{{\partial T}}{{\partial r}}} \right) - \hat {\alpha }{S_c}{{ex}}{{{p}}^{ - {\rm E}}}\left( {1+n{\beta _t}T} \right)\left( {1+{\rm E}{\beta _t}T} \right)=0.$$

The dynamics of motile microorganisms are described by the following formula:

30 $$\frac{1}{r}\frac{\partial }{{\partial r}}\left( {r\frac{{\partial N}}{{\partial r}}} \right) - P_{e} \left( {\left( {N + 1} \right)\frac{{\partial ^{2} C}}{{\partial r^{2} }} + \frac{{\partial N}}{{\partial r}}\frac{{\partial C}}{{\partial r}}} \right) = 0.$$

The flow volume calculated in the wave (moving) frame is represented using non-dimensional variables as

31 $$q = \mathop \smallint \limits_{0}^{{r_{2} }} wrdr.$$

The dimensionless form of the boundary conditions is reduced to:

$$u=2\pi \left( {\epsilon +\zeta } \right){{sin}}2\pi z,~~~~w= - 1 - 2~\alpha ~\zeta ~{{cos}}2\pi z - {\gamma ^{{*}}}\left( {1 - {S_{Tb}}{{\left( {\frac{{\partial w}}{{\partial r}}} \right)}^2}} \right)\frac{{\partial w}}{{\partial r}},~~$$

32 $$T=C=N=0~~~~~at~~~~r={r_2}=r\left( z \right)+{H_1},$$

33 $$u=0,~~~~\frac{{\partial w}}{{\partial r}}=\frac{{\partial T}}{{\partial r}}=\frac{{\partial C}}{{\partial r}}=~\frac{{\partial N}}{{\partial r}}=0~~~~~at~~~~r=0,~~~~$$

where

34 $${H_1}\left( {{{z}};{{t}}} \right)=1+({\epsilon _1}+\zeta ){{cos}}2\pi z - {\epsilon _2}{{co}}{{{s}}^4}\left( {\frac{\pi }{{{\lambda ^*}}}\left( {z+t} \right)} \right).$$

The non-dimensional representation of $R\left( z \right)$ is given by:

35 $$R\left( z \right)=\left\{ {\begin{array}{*{20}{l}} {1 - {m_1}\left( {z+L+d} \right)}&{~~~~ - {{L}} \leqslant {{z}}< - {{{z}}_0},} \\ {1 - {m_1}\left( {z+L+d} \right) - \frac{{h{{cos}}\phi }}{2}\left[ {1+{{cos}}\frac{{\pi z}}{{{L_0}}}} \right]}&{~~~~ - {{{z}}_0} \leqslant {{z}} \leqslant {{{z}}_0},} \\ {1 - {m_1}\left( {z+L+d} \right)}&{~~~~{{{z}}_0}<{{z}} \leqslant {{d}}.} \end{array}} \right.$$

Navier-Stokes calculations’ time-varying variables are frequently disregarded in extended-wavelength approximations due to viscosity and pressure forces predominate over fluid inertia, making the flow almost quasi-steady. However, the flow behavior gets more complicated when the tube or channel walls are rough and cilia-covered. Over time, the fluid interacts with further turbulence, eddies, and local perturbations brought about by surface roughness and cilia activity. Heat, solute, and microbial movement, velocity profiles, wall shear stress, and other time-dependent variations are all impacted by these phenomena. Additionally, at the fluid-wall interface, the cilia increase friction and momentum exchange, which amplifies the impact of time-dependent perturbations on the flow dynamics. Consequently, time is reintroduced into the governing equations via the roughness and cilia factors together. A major innovation of the current work is the coupling of surface roughness with active cilia motion, underscoring its importance for simulating realistic peristaltic flows in biological systems. In order to get approximate results for velocity, temperature, concentration, and microbe patterns, the equations associated with the model are solved in the subsequent section by using a perturbation approach and handling the tiny parameter $\delta$ using the HPM methodology.

## Solution procedure

When paired with the conventional perturbation method, the Homotopy Perturbation Method (HPM) offers a reliable and effective framework for resolving the current nonlinear equations. For highly nonlinear or coupled systems in particular, this hybrid technique resolves frequent issues with traditional perturbation approaches. HPM was chosen because it provides accurate analytical approximations with relatively simple computations for strongly nonlinear problems, including those with time-dependent boundary conditions and coupled effects such as cilia motion and wall roughness. Unlike traditional perturbation methods, HPM does not require a small parameter and converges rapidly to the solution, making it particularly suitable for our model of Sutterby nanofluid flow in stenosed arteries. As there are alternative analytical or numerical tools for such a problem, such as the ADM or the finite difference method, the HPM provides a compromise between the analytical and computational aspects, allowing us to find explicit formulas for the relevant physical fields (velocity, pressure, heat transfer) while maintaining the compatibility with the complex physiological aspects considered in the problem.

To build the homotopy and methodically provide approximate solutions, HPM introduces parameter $\:{p}_{h}$, which ranges from 0 to 1^47^. One of HPM’s main advantages is that it can generate fast convergent series without the need for modest physical parameters. By analyzing subsequent approximations, the convergence of the solution has been confirmed in the current investigation, demonstrating that the first two to three terms already yield extremely accurate findings. In line with other research, this validates HPM’s dependability for challenging fluid dynamics issues^47^. This method respects the dimensionless boundary conditions (32) and (33) while applying HPM to the nonlinear differential Eqs. (27–31). Additionally, the technique can properly depict all coupled effects in the peristaltic stenosed artery by capturing the interactions of Sutterby nanofluid, cilia dynamics, surface roughness, and motile bacteria.

36 $$\begin{aligned} H\left( {{{w}},{p_h}} \right) & =L\left( {{w}} \right) - L\left( {{w_0}} \right)+{p_h}L\left( {{w_0}} \right) \\ ~ & \;\;\;+~{p_h}\left\{ {\begin{array}{*{20}{c}} {\frac{{ - ~{S_{Tb}}}}{r}\frac{\partial }{{\partial r}}\left\{ {r{{\left( {\frac{{\partial w}}{{\partial r}}} \right)}^3}} \right\} - {F_r}\left( {{w^2}+2w} \right)+{G_r}T - {R_N}C - {R_b}N} \\ { - \frac{{~{\alpha _e}{H^2}}}{{~\alpha _{e}^{2}+\beta _{e}^{2}}}w - \frac{1}{{{D_{Ma}}}}\left( {w - {S_{Tb}}{{\left( {\frac{{\partial w}}{{\partial r}}} \right)}^2}\left( {w+1} \right)} \right)~} \end{array}} \right\}, \\ \end{aligned}$$,

37 $$\begin{aligned} H\left( {T,{p_h}} \right) & =L\left( {{T}} \right) - L\left( {{T_0}} \right)+{p_h}L\left( {{T_0}} \right) \\ & \;\;\;+\left\{ \begin{gathered} \frac{{{D_u}{P_r}}}{{1+{R_n}}}\frac{1}{r}\frac{\partial }{{\partial r}}\left( {r\frac{{\partial C}}{{\partial r}}} \right)+\frac{{{B_r}}}{{1+{R_n}}}\left( {\frac{{~{H^2}}}{{{{~}}\alpha _{e}^{2}+\beta _{e}^{2}}}+\frac{1}{{{D_{Ma}}}}\left( {1 - {S_{Tb}}{{\left( {\frac{{\partial w}}{{\partial r}}} \right)}^2}} \right)} \right){(w+1)^2} \hfill \\ +\frac{{{B_r}}}{{1+{R_n}}}\left( {1 - {S_{Tb}}{{\left( {\frac{{\partial w}}{{\partial r}}} \right)}^2}} \right){\left( {\frac{{\partial w}}{{\partial r}}} \right)^2}+\frac{{{N_b}}}{{1+{R_n}}}\frac{{\partial C}}{{\partial r}}\frac{{\partial T}}{{\partial r}}+\frac{{{N_t}}}{{1+{R_n}}}{\left( {\frac{{\partial T}}{{\partial r}}} \right)^2} \hfill \\ \end{gathered} \right\}, \\ \end{aligned}$$

38 $$\begin{aligned} H\left( {C,{p_h}} \right) & =L\left( C \right) - L\left( {{C_0}} \right)+{p_h}L\left( {{C_0}} \right)+~ \\ ~ & ~\;\;\;+{p_h}\left[ {\frac{{{N_t}}}{{{N_b}r}}\frac{\partial }{{\partial r}}\left( {r\frac{{\partial T}}{{\partial r}}} \right) - \hat {\alpha }{S_c}{e^{ - {\rm E}}}\left( {1+n{\beta _t}T} \right)\left( {1+{\rm E}{\beta _t}T} \right)} \right], \\ \end{aligned}$$

39 $$H\left( {N,{p_h}} \right)=L\left( N \right) - L\left( {{N_0}} \right)+{p_h}L\left( {{N_0}} \right)~+{p_h}\left[ {1 - {P_e}\left( {\left( {N+1} \right)\frac{{{\partial ^2}C}}{{\partial {r^2}}}+\frac{{\partial N}}{{\partial r}}\frac{{\partial C}}{{\partial r}}} \right)} \right].$$

To obtain the first estimate for the speed ${w_0}$, we introduce the linear component$~L$ by doing the following:

$L=\frac{1}{r}\frac{\partial }{{\partial r}}\left( {r\frac{\partial }{{\partial r}}} \right) - \frac{{d{{P}}}}{{dz}} - {F_r} - \frac{1}{{{D_{Ma}}}} - \frac{{~{\alpha _e}{H^2}}}{{~\alpha _{e}^{2}+\beta _{e}^{2}}}$. As a result, the initial hypothesis ${w_0}$ is expressed as follows:

40 $${w_0}\left( {r,z;t} \right)={d_3}{r^2}+{d_4},$$

The velocity distribution corresponding to the primary inflow is described by ${{{w}}_0}$, which represents the baseline Hagen–Poiseuille flow before the onset of peristaltic motion. Similarly, the initial assumptions for the temperature field ${{{T}}_0}$ are captured through a linear approximation, providing a starting point for analyzing the effects of peristalsis, wall roughness, and cilia dynamics. The linear element${{~L}}=\frac{1}{{{r}}}\frac{\partial }{{\partial {{r}}}}\left( {{{r}}\frac{\partial }{{\partial {{r}}}}} \right)+\frac{{{Q}}}{{1+{{{R}}_{{n}}}}}$ is used to identify the primary theories put out for temperature ${{{T}}_0}$ as follows:

41 $${T_0}\left( {r,z;t} \right)= - \frac{Q}{{4(1+{R_{n)}}}}\left( {{r^2} - r_{2}^{2}} \right).$$

The linear component${{L}}=\frac{1}{{{r}}}\frac{\partial }{{\partial {{r}}}}\left( {{{r}}\frac{\partial }{{\partial {{r}}}}} \right) - \alpha {S_c}{e^{ - {\rm E}}}$ is used to show the primary hypothesis for concentration${{{C}}_0}$ as follows:

42 $${C_0}\left( {r,z;t} \right)=\frac{{\alpha {S_c}{e^{ - {\rm E}}}}}{4}\left( {{r^2} - r_{2}^{2}} \right).$$

The linear component $L=\frac{1}{r}\frac{\partial }{{\partial r}}\left( {r\frac{\partial }{{\partial r}}} \right) - {P_e}$ is used to show the primary hypothesis for microorganism ${N_0}$ as follows:

43 $${N_0}\left( {r,z;t} \right)=\frac{{{P_e}}}{4}\left( {{r^2} - r_{2}^{2}} \right).$$

This fundamental assumption allows for the development of the profiles $w,~T,~C$, and *N* as:

44 $${{\boldsymbol{\Lambda}}}={{{\boldsymbol{\Lambda}}}_0}+{p_h}~{{{\boldsymbol{\Lambda}}}_1}+...,$$

where ${\Lambda _0}$ signifies the zero order of ${p_h}$. While$~{\Lambda _1}$ signifies the first one.

The final profiles for velocity, temperature, concentration, and motile microorganism distribution, corresponding to ${p_h}=1$, are obtained as follows, representing the fully developed state after applying the H PM.

45 $${{W}}\left( {{{r}},{{z}}} \right)=\frac{{{d_5}}}{{36}}{r^6}+\frac{{{d_6}}}{{16}}{r^4}+\left( {\frac{{{d_7}}}{4}+{d_3}} \right){r^2}+{d_8}++{d_4},$$

46 $$T\left( {r,z} \right)=\frac{{{d_9}}}{{64}}{r^8}+\frac{{{d_{10}}}}{{36}}{r^6}+\frac{{{d_{11}}}}{{16}}{r^4}+\left( {\frac{{{d_{12}}}}{4} - \frac{Q}{{4(1+{R_{n)}}}}} \right){r^2}+{d_{13}}+\frac{{Q~r_{2}^{2}}}{{4(1+{R_{n)}}}},$$

47 $$C\left( {r,z} \right)=\frac{{{d_{14}}}}{{36}}{r^6}+\frac{{{d_{15}}}}{{16}}{r^4}+\left( {\frac{{{d_{16}}}}{4}+\frac{{\alpha {S_c}{e^{ - {\rm E}}}}}{4}} \right){r^2}+{d_{17}} - \frac{{\alpha {S_c}{e^{ - {\rm E}}}}}{4}r_{2}^{2},$$

48 $$N\left( {r,z} \right)=\frac{{{d_{18}}}}{{16}}{r^4}+\left( {\frac{{{d_{19}}}}{4}+\frac{{{P_e}}}{4}} \right){r^2}+{d_{20}} - \frac{{{P_e}}}{4}r_{2}^{2}.$$

In the Homotopy Perturbation Method (HPM), the solution is given as a series: ${{\boldsymbol{\Lambda}}}={{{\boldsymbol{\Lambda}}}_0}+{p_h}~{{{\boldsymbol{\Lambda}}}_1}+....$.The solution is considered convergent if the successive terms $~{{{\boldsymbol{\Lambda}}}_{n+1}}$ are sufficiently small in comparison to the previous terms. To ensure the robustness and reliability of the solution, a convergence criterion has been applied. The iterations are continued until the difference between successive approximations of all the key variables, such as velocity, pressure, temperature, and concentration, is considered negligible.

The volume flow rate directly influences the evaluation of $G\left( z \right)$, which represents the flow characteristics along the axial direction. By combining Eqs. (22) and (31), the impact of the peristaltic motion, wall roughness, and cilia dynamics on the volumetric transport can be systematically accounted for:

49 $${{\boldsymbol{\Xi}}}=q+{r_2}{(z)^2},$$

from Eq. (23)

50 $$\hat {Q}={{q}}+\frac{1}{2}{\left( {{{R}}\left( {{z}} \right)+} \right)^2}+\frac{1}{4}{\left( {{\epsilon _1}+{{\boldsymbol{\upzeta}}}} \right)^2}+{\epsilon _2}{{co}}{{{s}}^4}\left( {\frac{\pi }{{{\lambda ^*}}}z} \right)\left[ {\frac{{{\epsilon _2}}}{2}{{co}}{{{s}}^4}\left( {\frac{\pi }{{{\lambda ^*}}}z} \right) - R\left( z \right) - 1} \right].$$

Thus, it is possible to calculate the solution of $G\left( z \right)$ as:

51 $$G\left( z \right)=\frac{{dP}}{{dz}}=\frac{{16}}{{r_{2}^{2}\left( {r_{2}^{2} - 8{d_1}} \right)}}\left\{ { - {d_2}+\hat {Q} - \frac{1}{2}{{\left( {{{R}}\left( {{z}} \right)+} \right)}^2} - \frac{1}{4}{{\left( {{\epsilon _1}+{{\boldsymbol{\upzeta}}}} \right)}^2}+{\epsilon _2}{{co}}{{{s}}^4}\left( {\frac{\pi }{{{\lambda ^*}}}z} \right)\left[ {\frac{{{\epsilon _2}}}{2}{{co}}{{{s}}^4}\left( {\frac{\pi }{{{\lambda ^*}}}z} \right) - ~~R\left( z \right) - 1} \right]} \right\}.$$

The constants$~{d_1},$ ${d_2},$….$,~{{and}}~{d_{20}}$have been determined; nevertheless, for brevity and clarity, their explicit expressions are omitted from this paper.

An elevation in the pressure of liquid across a tube or apparatus, which can result from a variety of factors relating to the flow design, is referred to as a pressure rise. This effect is mainly caused by the channel walls’ compression and relaxation, which raises the internal pressure in peristaltic flows. Maintaining the effectiveness and security of engineering and biological systems requires an understanding of the processes and consequences of pressure increase. In order to maximize performance and prevent unfavorable effects, designers, researchers, and practitioners closely examine these pressure changes. The pressure rise $\Delta P$^22^ in the dimensionless formulation can be established by:

52 $$\Delta P=\mathop \smallint \limits_{{ - L}}^{d} G\left( z \right)dz=\mathop \smallint \limits_{{ - L}}^{{ - {z_0}}} G\left( z \right)dz+\mathop \smallint \limits_{{ - {z_0}}}^{{{z_0}}} G\left( z \right)dz+\mathop \smallint \limits_{{{z_0}}}^{d} G\left( z \right)dz.$$

This is given as the frictional force $\Delta F$. As the roughness of the walls increases, there is a corresponding increase in resistance. Dimensionless expression: As already seen in the expression of dimensionality loss, we have the expression for the friction force $\Delta F$^22^, which is given as:

53 $$\Delta F=\mathop \smallint \limits_{{ - L}}^{d} r_{2}^{2}G\left( z \right)dz=\mathop \smallint \limits_{{ - L}}^{{ - {z_0}}} r_{2}^{2}G\left( z \right)dz+\mathop \smallint \limits_{{ - {z_0}}}^{{{z_0}}} r_{2}^{2}G\left( z \right)dz+\mathop \smallint \limits_{{{z_0}}}^{d} r_{2}^{2}G\left( z \right)dz.$$

Because these integrals are difficult, we compute them using numerical techniques.

Skin friction ($S{k_f}$) refers to the tangential stress applied by the fluid. The dimensionless skin-friction coefficient for the Sutterby structure can be established as:

54 $$S{k_f}={\left. {\left( {1 - {S_{Tb}}{{\left( {\frac{{\partial w}}{{\partial r}}} \right)}^2}} \right)\left( {\frac{{\partial w}}{{\partial r}}} \right)} \right|_{r={r_2}\left( {z;t} \right)}}.$$

${{Nu}}$ represents the Nusselt factor, which means the relative effectiveness of the convective heat transfer with respect to conductive heat transfer. It gives information about the efficiency of the heat exchange between blood and the arterial wall. ${{Nu}}$ dimensionless Nusselt form is given by^11,12^:

55 $${{Nu}}= - {\left. {\frac{{\partial T}}{{\partial r}}} \right|_{r={r_2}\left( z \right)}}.$$

The Sherwood number (${{Sh}}$) is convective mass transfer relative to diffusion. It indicates effectively substances such as oxygen, nutrients, or therapeutic agents which are transported between the blood and the arterial wall. The dimensionless Sherwood number (${{Sh}}$)^11,12^ is shown as:

56 $${{Sh}}= - {\left. {\frac{{\partial C}}{{\partial r}}} \right|_{r={r_2}\left( z \right)}}.$$

The local density of motile microorganisms can be expressed as^11,12^:

57 $${{{N}}_{LD}}= - {\left. {\frac{{\partial N}}{{\partial r}}} \right|_{r={r_2}\left( z \right)}}.$$

## Findings and discussion

Mathematica software (version 12.0.0.0) was utilized to describe the numerical computations and graphical representations. The behavior of axial flow velocity (*W*), variation in temperature (*T*), concentration (*C*), motile microbe (*N*), force friction ($\Delta F$), as well as pressure rise (ΔP), was discussed.

The chosen values of the factors were depicted relied on physical state and consistency with previously validated theoretical and experimental investigations stated in the literature; see Refs^10,18,20,21,33,37,46^. This range is selected as: $\zeta =0.2$, $\alpha =0.3$, ${H^2}=1.2$, ${ \in _1}=0.3$, ${ \in _2}=0.2$, ${F_r}=0.5$, ${D_{Ma}}=0.1$, ${G_r}=5$, ${R_N}=3$, ${R_b}=2.5$, ${N_t}=3$, ${N_b}=2$, $\hat {\alpha }=0.2$, $L=2$, ${z_0}=0.7$, $d=2$, ${\beta _e}=0.01$, ${\gamma ^{{*}}}=0.1$, $\phi =0.05$, $h=0.1$, ${R_n}$=2,$~\hat {Q}=0.8$, $Q=0.2$, ${S_c}=0.5$, ${D_u}=0.1$, ${P_r}=1.2$, ${B_r}=2.5$, ${\beta _t}=0.01$, $n=0.1$, ${{t}}=1$,${{~}}{{{\boldsymbol{\uplambda}}}^*}=0.1$,${{~}}{{{\boldsymbol{\upalpha}}}_e}=0.1$, ${P_e}=0.5$ and ${{{S}}_{Tb}}=0.4.{{~}}$.

The principal results are presented in Figs. 2, 3, 4, 5, 6, 7, 8, 9 and 10 arranged as follows.

### Velocity characteristics

**Fig. 2:**
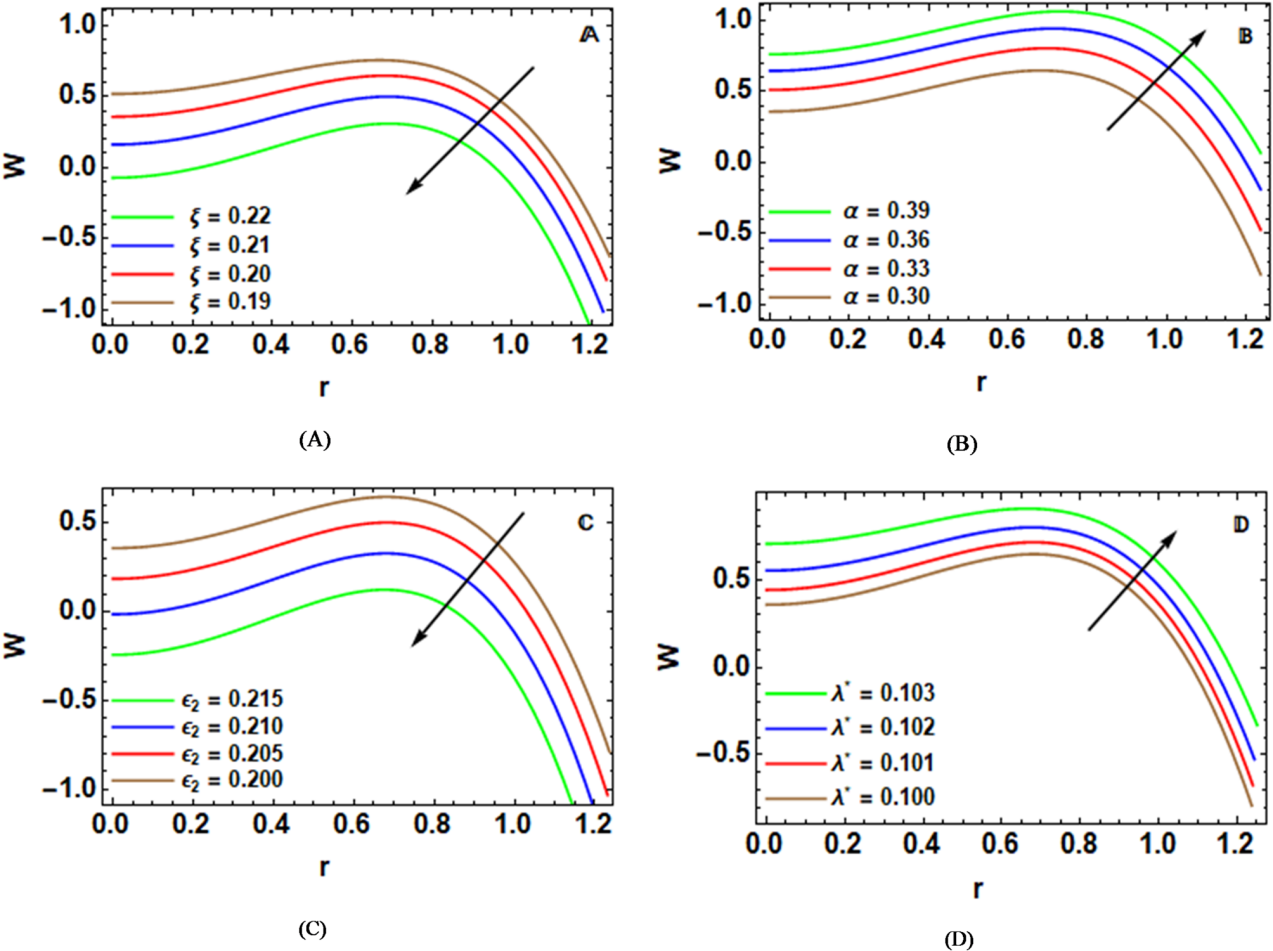
(A) Depicts alternative of $w\left( r \right)$ corresponding to various ranges of $\xi .~$ (B) Depicts alternative of $w\left( r \right)$ corresponding to various ranges of${{~~\boldsymbol{\upalpha}}}.{{~~}}$ (C) Depicts alternative of $w\left( r \right)$ corresponding to various ranges of${{~}}{\epsilon _2}.~$ (D) Depicts alternative of $w\left( r \right)$ corresponding to various ranges of ${{~}}{{{\boldsymbol{\uplambda}}}^*}$.

Figure 2-A shows how great the cilia are$~\xi$ on the axial speed $W\left( r \right)$. It describes the effective range of tiny fingerlike protrusions linked to the wall like hairs on skin of an arterial occlusive vessel. Shirt tail molecules and other tiny particles (like blood cells) floating in an eddy current that is slow enough cannot easily be brushed off a liquid surface. When it is increased, the change between the moving wall and the surrounding water takes on a material nature. As the cilia become longer, they go deeper into the work domain, which raises flow resistance and viscous drag higher still. Therefore, the rate of axial fluid flow is greatly diminished^46^. Flow deceleration becomes stronger when surface roughness and mild stenosis act together with cilia motion, leading to higher energy loss and reduced velocity. The velocity field shows both forward and backward motion, a typical feature of peristaltic flow caused by wall contraction and geometric restriction. These findings are useful in understanding mucus transport in airways, regulating blood flow in narrowed vessels, and improving mixing and flow control in microfluidic devices and drug delivery systems^23^.

Figure 2-B depicts that an increase in the degree of eccentricity during the elliptical motion of cilia increases the axial speed dispersion, W(r). However, surface irregularity, coupled with stenosis, resists the motion, yet the high degree of ciliary motion overcomes these hurdles by energizing the fluid. Shear, coupled with a reduced time to recover, directly increases the efficiency of the forward motion^24^. An interesting point is the sign reversal in the velocity profile, as peristaltic transport is characterized by negative values rather than positive ones. So, in the case of the transport process, positive values mean going forward due to the peristaltic traveling wave and the cilia activity that is mainly causing it; correspondingly, the negative values mean going backward due to the deformability of the vessel wall, roughness, and constrictions. These positive values are going up for eccentric values, and on the vessel wall, they turn to zero. When cilia sit off-center, real-world effects show up in medical and bio-inspired designs. Looking at living bodies, this shift might explain better mucus removal, handling blood movement, or how fluids move across ciliated surfaces - especially when illness alters normal structures like in narrowed arteries. In built systems, such as lab-on-a-chip setups, adjusting artificial cilium position could make pumps work more smoothly, deliver medicine more reliably, while managing fast flows near uneven or blocked passages. So here’s how it breaks down - cilia placed off center, tiny surface imperfections, along with slight blockage in a channel, together shape how fluid moves through intricate living and engineered environments^25^.

The change in the longitudinal velocity $w\left( r \right)$ in relation to the wall roughness amplitude ratio ${\epsilon _2}$ is depicted in Fig. 2-C. The wall roughness amplitude ratio represents the normalized measure of surface irregularity height relative to the characteristic arterial radius, and it is a crucial parameter for capturing deviations from an ideal smooth boundary. In a peristaltic flow of a Sutterby nanofluid, wall roughness plays a dominant role in modifying shear distribution and flow resistance due to the fluid’s non-Newtonian rheological behavior. An increase in the wall roughness amplitude ratio results in a reduction of the axial velocity $w\left( r \right)$, even in the presence of cilia-driven motion and mild stenosis. Physically, larger surface irregularities intensify frictional resistance and disrupt the organized momentum transfer generated by cilia beating. Although cilia tend to propel the fluid forward and mild stenosis alters the pressure gradient, enhanced roughness counteracts these effects by increasing energy dissipation and weakening the effective pumping capability of the peristaltic wave. In the case of a Sutterby fluid, elevated roughness magnifies local shear fluctuations, which can increase apparent viscosity in certain regions, further slowing down the flow^20^. The velocity profiles shown in the corresponding figure display both positive and negative velocity values, a characteristic feature of peristaltic transport. From an application standpoint. These are highly relevant for results of physiological blood flow analysis and biomedical device design. In biological systems, increased wall roughness can represent advanced atherosclerotic conditions, where excessive surface irregularities. However, blood transport is hindered by natural ciliary motion. Also, in engineering microfluidic applications, this highlights the importance of controlling surface texture in peristaltic pumps, artificial vessels, and drug delivery devices operating with non- Newtonian fluids. Proper correction of wall roughness is important for propulsion efficiency, in flow stability, and transport efficiency of complex peristaltic systems^21^.

The change in the longitudinal velocity $w\left( r \right)$ in relation to the wall roughness pitch ratio ${{{\boldsymbol{\uplambda}}}^*}$ appears in Fig. 2-D. This parameter controls how often surface irregularities occur in the flow direction and plays a vital role in creating near- wall hydrodynamics. An increase in ${{{\boldsymbol{\uplambda}}}^*}$ leads to an enhancement of the axial velocity, especially in the presence of cilia motion and mild stenosis. Physically, larger ${{{\boldsymbol{\uplambda}}}^*}$ values result in more spread out roughness elements, which result in less repeated fluid obstruction and allow cilia-driven movement to act more efficiently on the fluid. These findings align well with previous observations reported by Shukla et al.^22^. In technological systems, optimizing this parameter can improve the performance peristaltic micropumps, artificial blood vessels, drug delivery channels, and lab- on- a- chip devices that function with non-Newtonian fluids.

**Fig. 3:**
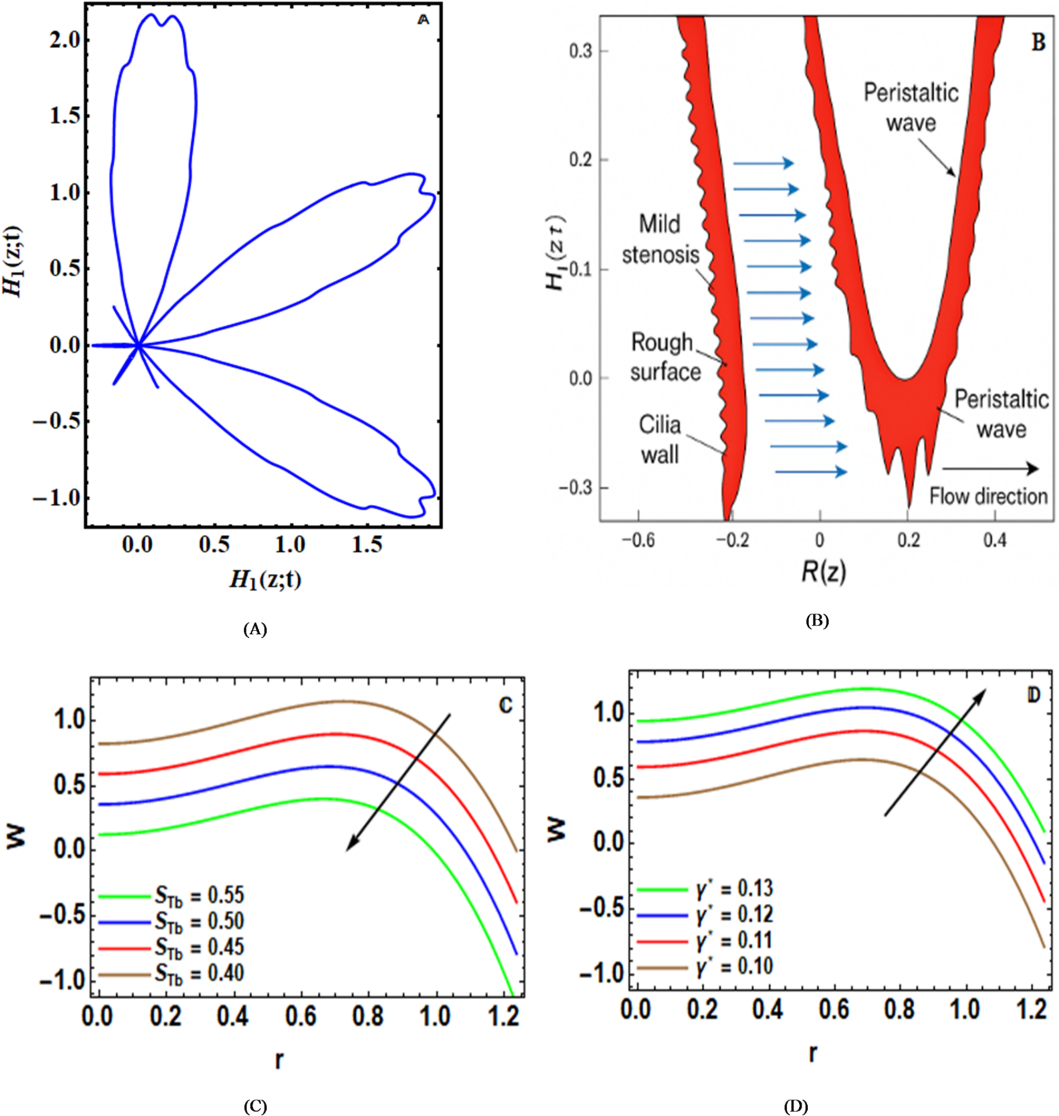
(A) Depicts PolarPlot for $\:{H}_{1}(z;t)$. (B) Depicts ParametricPlot among $\:{H}_{1}(z;t)$ and $\:R\left(z\right)$. (C) Depicts alternative of $\:w\left(r\right)$ corresponding to various ranges of $\:{{S}}_{Tb}.\:$ (D) Depicts alternative of $\:w\left(r\right)$ corresponding to various ranges of $\:{{\upgamma\:}}^{*}$.

A PolarPlot is a graphical determination of the position of the cilia wall, versus ${H_1}\left( {z;t} \right)$, changes if that position is shifted along the artery, as evidenced by Fig. 3-A. The diagram, moving up and down like peaks and troughs, indicates the maximum amount of the cilia position. At the end of the figure, the non-uniform surface generated due to the effect of the cilia and roughness encourages the sheer effect of interaction as well as the formation of microscopic vortices in the artery. This effect can be of significant importance in the context of biomedical applications. Further, the figure covers the entire range of negative and positive ${H_1}\left( {z;t} \right)$, illustrating the forward and backward motions of the cilia during the entire cycle of peristalsis. Every petal formed in the polar graph is the result of the wave crest. More elongated petals suggest an increased level of interaction. Therefore, in the context of the figure discussed above, the novelty of the current study comes as the verification in the form of the polar graph.

Figure 3-B investigates the spatial distribution of the radius of the artery along with the corresponding peristaltic wave transmission while the artery is stenosed with ciliated and rough-surfaced prognosis zones. The fluid is seen to accelerate locally as a direct consequence of the change in the cross-sectional flow area due to the stenosis of the artery, as is clearly illustrated along the center portion of the constriction due to the continuity principle. However, the fluid is seen to experience a greater shear as well as a depressed axial flow as a direct consequence of the abnormal velocities caused by the uniform surface velocities attributed to the presence of the nonsmooth surface activated by the extra strength of the viscous fluid, accountable for the increaseddrag force. Here, the stenosis and the passageway wall irregularities are factors influencing the peristaltic waves, which are seen as the periodic changes in the flow of the blood within the artery. The peristaltic pumping action is subsequently dynamically adjusted in line with the changes in the walls, as measured by the functions $\eta \left( {{{Z}};{{t}}} \right)$. It is evident from the figure in the paper that the link between the peristaltic action in the arteries, the makeup of the walls inside the arteries, and the form of the arteries is very complicated, especially when considering the significant role that the effects of the cilia movement and the roughness of the arteries have on the flow velocity of the Sutterby nanofluid in the arteries that have been narrowed in this way.

The Sutterby factor’s ${S_{Tb}}$ is also influenced on w(r), as shown in Fig. 3-C. By raising ${S_{Tb}}$, the material becomes more shear-thinning, making the hydrodynamic resistance more significant, particularly in the reduced cross-section of the arteries, thus lowering the velocity of the fluid. The explanation can be given by the more significant resistance of the structure of the fluid to deformation during the movement of the peristalsis mechanism, thus needing more pumping to move the fluid. Fewer non-Newtonian effects of the fluid make the movement smoother, particularly when the ${S_{Tb}}$ is reduced. These results have great biological relevance to the areas of the body where the blood is more rheologically responsive to shear. Variations in shear-thinning properties can directly affect transport efficiency, while controlled manipulation of the fluid’s rheology, nanofluid formulations, or medical equipment affected by possible route to the control of flow performance in complicated vascular settings. These findings are in agreement with the observations reported by Chinnasamy et al.^7^.

Figure 3-D emphasizes the efficacy of the slip element ${\gamma ^{{*}}}$ on $w\left( r \right)$. The slip factor can quantify the degree of velocity at the wall, which is deduced from the no-slip condition. It allows the fluid to slide along the boundary. Physically, a higher slip element reduces the viscous resistance near the wall. Therefore, it enables the fluid to move more freely, resulting in an increase in axial velocity. There are many applications, such as micro- and nano-scale flows, glycocalyx layers, ciliated endothelium, and lubricated arterial surfaces.

**Fig. 4:**
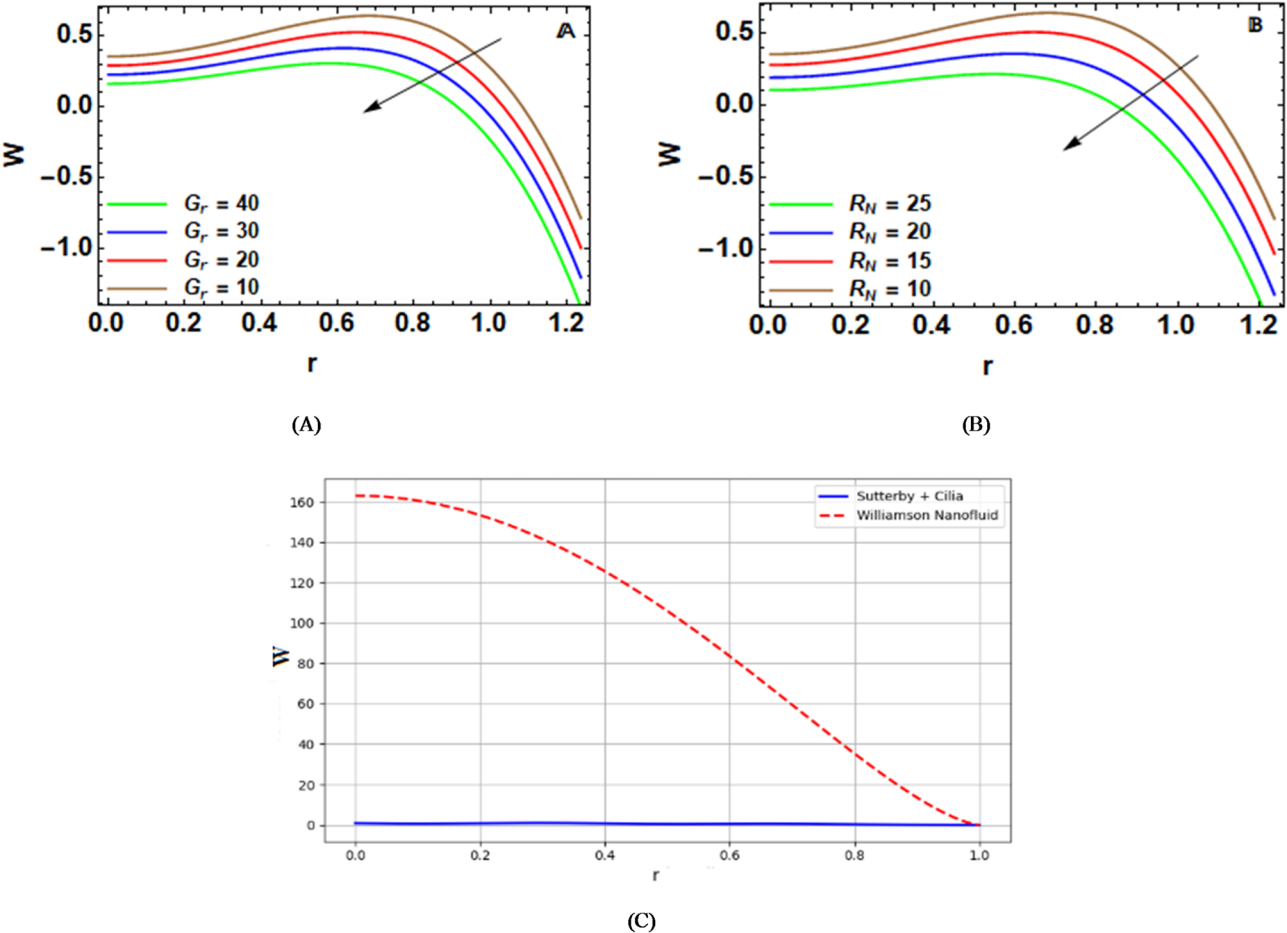
(A) Depicts alternative of $\:w\left(r\right)$ corresponding to various ranges of $\:\:{{G}}_{r}$. (B) Depicts alternative of $\:w\left(r\right)$ corresponding to various ranges of$\:\:\:{{R}}_{N}$. (C) Velocity comparison $\:w\left(r\right)$between this paper (Sutterby with cilia) and Mostapha^48^(Williamson without cilia).

The effect of the thermal Grashof factor ${G_r}$ on $w\left( r \right)$ profile is shown in Fig. 4-A. This factor is a comparison of buoyancy forces generated by a physical phenomenon of a non-uniform temperature pattern to viscous forces. Also, it is the effects of a variation in the density of a substance as a result of its own heat expansion in a fluid motion pattern in a micro-flow scenario. To be more technical, an increase in the physical effects produced by ${G_r}$ will result in a buoyancy effect produced by a gradient pattern in the fluid motion. Therefore, this can create resistance in the fluid motion or a halt, or a decrease in the actual velocity of a fluid substance in a micro-flow drafting pattern or scenario. It can be utilized in various practical applications, such as in hyperthermia therapy, in a microfluid device, in nanofluid cooling systems, and in an artificial vascular scenario, to name a few.

The impact of the nanoparticles’ Grashof ${R_N}$ on $w\left( r \right)$ is depicted in Fig. 4-B. It can determine the value of the buoyancy forces with respect to the viscous ones. Specifically, ${R_N}$ is a physical phenomenon that compares the effects produced by the change in the nanoparticles’ concentration with the viscous ones. Thus, ${R_N}$ may be regarded as a representation of the capacity of the change in the nanoparticles’ concentration to produce the phenomenon of natural convective current. As the value of ${R_N}$ is higher, the effects of buoyancy are enhanced; thus, the movement of the fluids is reduced as a result of the increase in the local resistances opposing the movement. In other words, the higher the nanoparticles’ concentration or the effects created by buoyancy, the lower the value of the fluid’s movement. There are a great number of uses, such as drug delivery systems, hyperthermia therapy, microvascular cooling, in lab-on-chip, and microfluidic devices.

The Sutterby Nanofluid with a ciliated boundary was also compared with the Williamson Nanofluid via a stenosed artery under peristaltic and MHD flow^48^. Figure 4-C depicts this comparison. The velocity profile of Sutterby Nanofluid is extremely low, with values between [-1,+1], while its velocity oscillates with minor fluctuations near its boundary with the artery. This is clearly because of hydrodynamic resistances arising from hydrodynamics and ciliated walls. On the other hand, Williamson Nanofluid’s velocity distribution far exceeds this with a maximum velocity of 163, and is very smooth and parabolic with values strictly along its axis of symmetry and everywhere positive. This stark difference between the two velocity distributions can again be physically justified as arising because of stronger shear-thinning of Sutterby Nanofluid at low rates of shear, causing increased viscosity near its artery boundary, and also due to its cilia. Additionally, both Nanofluid models are subject to Hall and ion-slip currents, and in this case of Sutterby Nanofluid with its ciliated boundary, these two major forces of magnetohydrodynamics also enhance the retardation of its flow.

Table 1. provides a comparison of the axial velocity characteristics between the present study, which investigates Sutterby nanofluid flow with a ciliated endothelial lining and dynamic wall roughness, and the study on Williamson nanofluid through a stenosed artery^48^. In the present study, the cilia introduce local micro-obstructions, resulting in bidirectional velocities and a reduction in net forward flow, whereas in the Williamson study, the absence of cilia allows purely forward flow with significantly higher velocities. Regarding fluid rheology, Sutterby fluid exhibits stronger shear-thinning at low shear rates, increasing effective viscosity near the wall and reducing velocity, while Williamson fluid shows milder shear-thinning, permitting higher peak velocities. Both studies indicate that wall roughness decreases the critical pumping velocity; however, in the present study, the time-dependent and dynamic roughness interacts with cilia and magnetic effects, generating complex backflows. Finally, although Hall and ion-slip effects influence velocity profiles in both models, their interaction with cilia in the present study further amplifies flow retardation and nonlinear behavior.

### Temperature structure

**Fig. 5:**
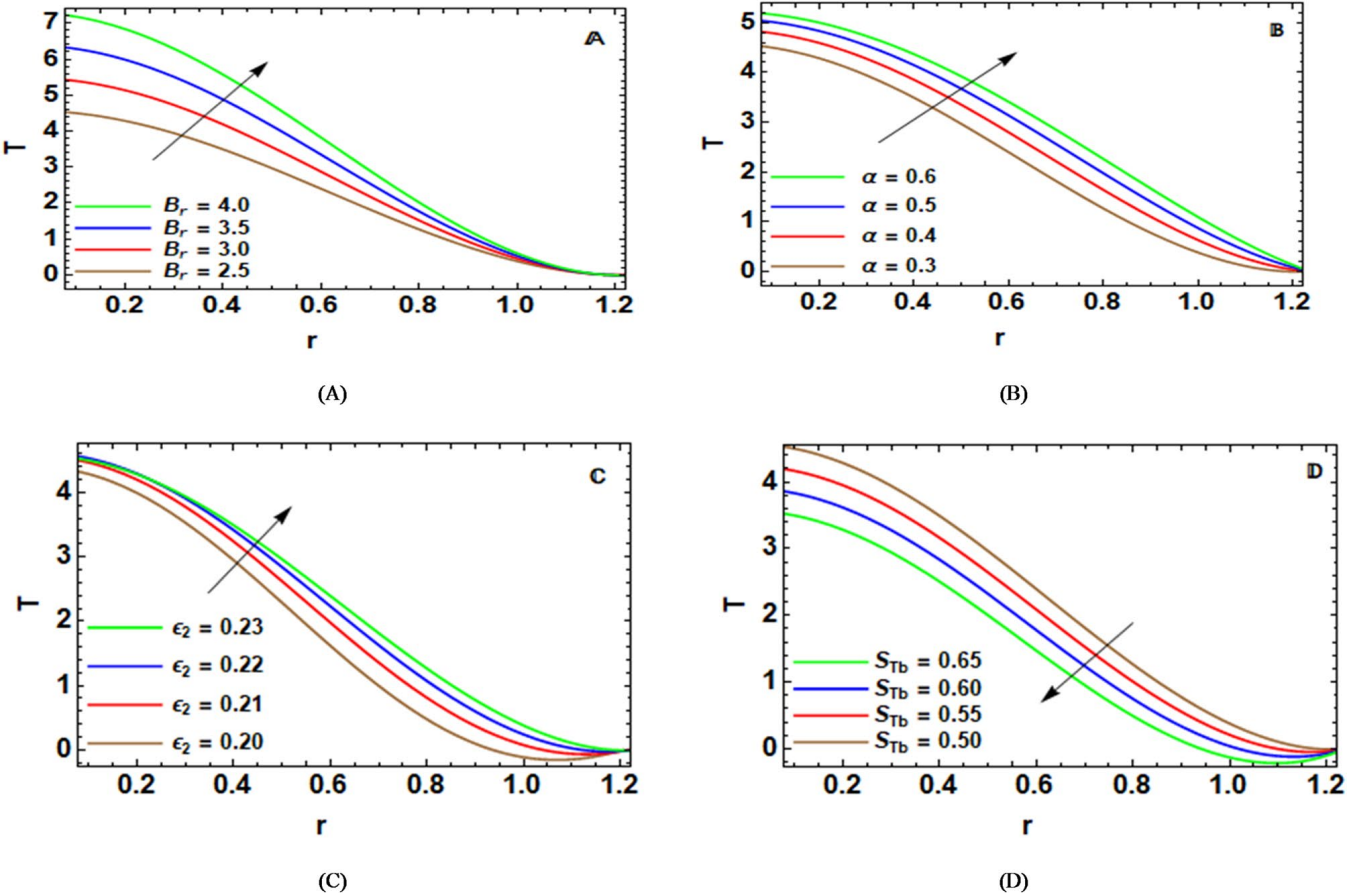
(A) Depicts alternative of $\:T\left(r\right)$ corresponding to various ranges of $\:{B}_{r}$. (B) Depicts alternative of $\:T\left(r\right)$ corresponding to various ranges of $\:{\upalpha\:}$. (C) Depicts alternative of $\:T\left(r\right)$ corresponding to various ranges of $\varepsilon _{2}$. (D) Depicts alternative of $\:T\left(r\right)$ corresponding to various ranges of $\:{S}_{Tb}$.

Figure 5-A shows the efficacy of Brickman numeral ${B_r}$ on $T\left( r \right)$. It defines the extent to which viscous dissipation enhances the conductive heat transfer in the fluids. In addition, it is the conversion of mechanical energy to thermal energy via the inner friction. There exists a distinct increase in the temperature distribution with an increase in the value of ${B_r}$. A higher value of ${B_r}$ exists alongside a higher viscous heating effect. This happens due to higher values of ${B_r}$ with higher values of shear stress. In turn, this produces an increase in temperature within the fluids. In a physical sense, the increased temperature for higher values of ${B_r}$ indicates an increase in the amount of thermal energy generated from the mechanical energy supplied by the peristaltic pumping action. In particular, this increase manifests itself for shear-thinning fluids consisting of Sutterby fluids with viscosity dependent on the magnitude of the shear rate. ${B_r}$ arganizes the viscous heating of blood flow, affecting tissue temperatures, biochemical reactions, hyperthermia treatment, and nanoparticle drug delivery in stenosed blood vessels.

The implementation of the eccentricity of the elliptical beating pattern of cilia $\alpha$ on $T\left( r \right)$ is demonstrated in Fig. 5-B. Eccentricity increases ciliary motion’s asymmetry and amplitude, which improves fluid mixing and local shear rates close to the ciliated wall. Stronger viscous dissipation from this increased shear results in more mechanical energy being converted to heat energy. As a result, when the eccentricity increases, the fluid temperature rises. Physically speaking, greater eccentric ciliary motion increases frictional heating by encouraging aggressive contact between the fluid layers and the wall, particularly when non-Newtonian effects and surface roughness are present. This temperature increase can alter reaction kinetics, impact nanoparticle transport and activation, and change heat transfer to vascular tissues in biomedical settings, underscoring the significance of ciliary beating properties in thermal control of peristaltic and cilia-driven flows.

The result of the influence of ${\epsilon _2}$on $T\left( r \right)$ is presented in Fig. 5-C. The increment of ${\epsilon _2}$ will increase fluid-wall interaction significantly because it will increase viscous dissipation and Joule heat effects, which may increase temperature in the fluid. The amplitude of surface roughness has a significant influence on regulating heat enhancement and flow direction in many applications of interest in heat transfer, drug delivery systems, etc., which are of great importance in various applications in natural flows. The results of this study are in total compliance with the preliminary findings of Mostapha et al.^47^.

The behavior of radial temperature $T\left( r \right)$ varies depending on different values of Sutterby factor ${S_{Tb}}$, which has been demonstrated in Fig. 5-D. An increase in ${S_{Tb}}~$produces a decrease in the values of viscosity in the area of high shearing, which finally reduces the temperature by viscous dissipation. By performing a reducing heat analysis through shearing and disturbed flow, we may understand this scenario. There may be more applications in the area of biomedical engineering. Thus, predictions may arise from blood flow and in drug delivery systems.

### Concentration and motile microorganisms

**Fig. 6:**
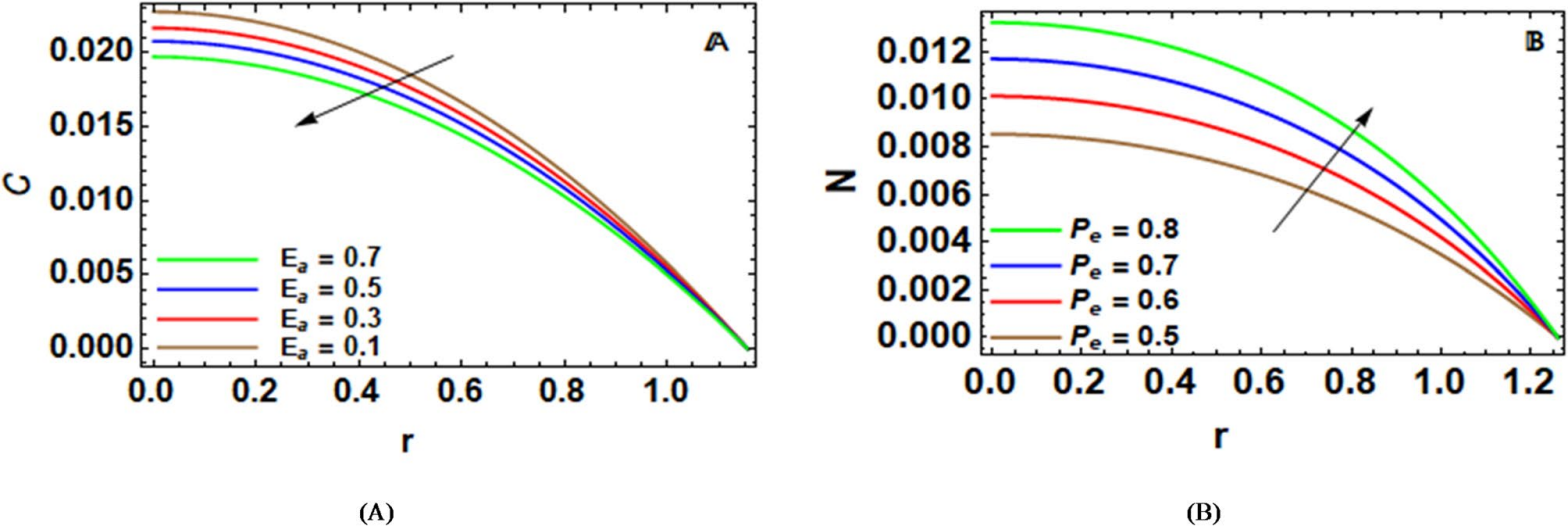
(A) Depicts alternative of $\:C\left(r\right)$ corresponding to various ranges of $\:{{{\rm\:E}}}_{a}.\:$ (B) Depicts alternative of $\:N\left(r\right)$ corresponding to various ranges of $\:{P}_{e}.$

Figure 6-A indirectly talks about the impact of activation energy $~{{{{\rm E}}}_a}$ on $C\left( r \right)$. It is the energy that will be supplied for the fluid transfer that will definitely happen in the fluid of interest. When ${{{{\rm E}}}_a}$ varies, less mass transfer will take place, hence decreasing concentration in all the fluids of interest. This physically implies that an increase in this energy will decrease concentration because of a slower reaction in the diffusion of the molecules, thus decreasing concentration. The implications of this equation are of great benefit in biomedical applications, specifically in controlling agents, optimizing nutrients, and transporting solutes in stenosed arteries that contain ciliated endothelium.

Figure 6-B exhibits a variance in motile microbe amount $N\left( r \right)$ over several biological migration phases, which is represented by the Peclet factor ${P_e}$. The ratio of advective to diffusive transport in a fluid system is represented by the Peclet factor ${P_e}$. The transfer of microorganisms along the flow direction is enhanced when advection predominates over diffusion, as shown by a greater ${P_e}$. Because advective motion transports microorganisms more efficiently and overcomes the spreading effect of molecular diffusion, the concentration of bacteria increases as ${P_e}$ increases. Physically, this happens because increased advection causes microorganisms to travel more quickly, which enables them to gather in areas downstream of the flow. This impact is enhanced by the presence of cilia, which provide advantageous pathways that direct bacteria down the peristaltic channel by causing localized motion and mixing. Microorganisms’ spatial distribution is further impacted by wall roughness and stenosed peristaltic amplitude, which create recirculation zones that momentarily trap microorganisms. Applications in biomedicine and engineering, such as managing targeted medication administration, improving microbial transport in microfluidic systems, and comprehending microorganism behavior in ciliated biological channels, might benefit from these insights. A thorough knowledge of the dispersion of microorganisms in complicated flows is made possible by the interplay of the Peclet number, cilia-induced motion, and flow geometry.

### Pressure rise and friction force

**Fig. 7:**
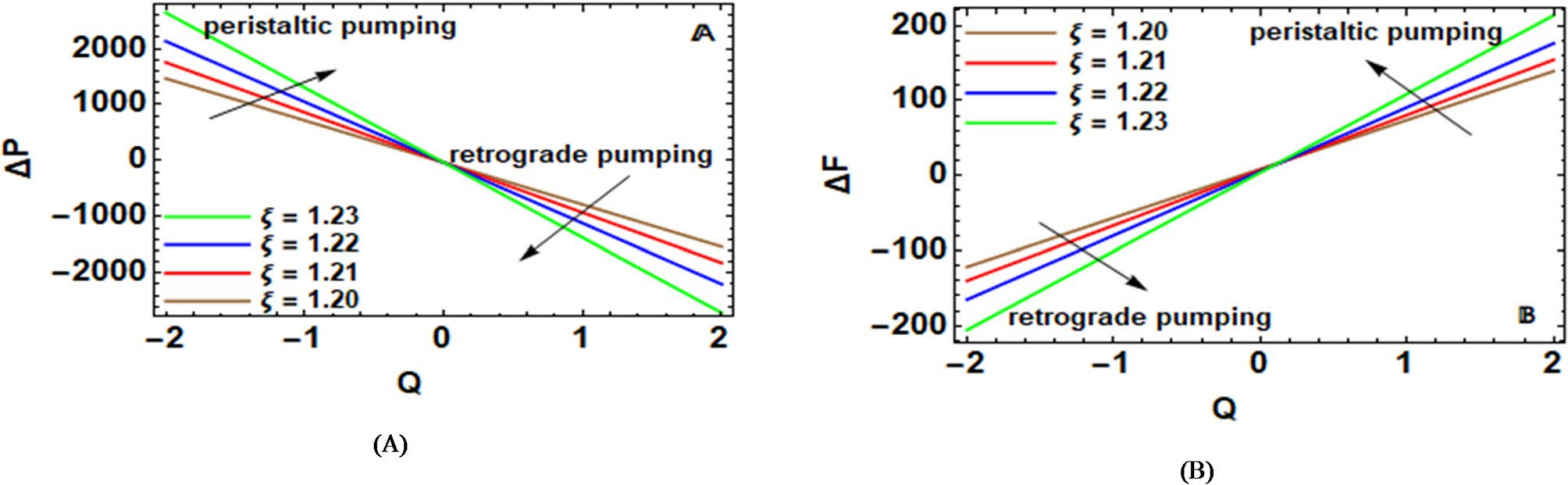
(A) Depicts alternative of $\:\varDelta\:P$ corresponding to various ranges of $\:\xi\:$. (B) Depicts alternative of $\:\varDelta\:F$ corresponding to various ranges of$\:\:\xi\:$.

The net pressure differential created over a single peristaltic wave wavelength to propel the fluid through the arterial section is represented by the pressure rise${{~\boldsymbol{\Delta}}}P$. Physically, it measures the peristaltic motion’s capacity to overcome stenosis, wall roughness, viscous resistance, and magnetic damping effects. In terms of physiology, the effort needed by artery walls to maintain blood flow through restricted areas is directly correlated with pressure rise. For varying values of the cilia-related parameter $\xi$, Fig. 7-A shows that the pressure rise ${{\boldsymbol{\Delta}}}P$ varies linearly with the volumetric flow rate. Peristaltic pumping, in which a positive pressure rise is necessary to sustain forward flow, is represented by the top area of the curves, whereas retrograde pumping, in which the flow is forced against the pressure gradient, is represented by the bottom region. For a constant flow rate, an increase in $\xi$ results in a decrease in the pressure rise, suggesting that increased ciliary activity adds more hydrodynamic resistance. This results from the intensification of near-wall shear stresses by cilia–fluid interactions, which raises viscous dissipation and lowers the effective pumping efficiency.

The net volumetric transfer caused by the peristaltic wave under a certain pressure situation is described by the friction force ${{\boldsymbol{\Delta}}}F$ force characteristic. This metric is essential for comprehending medication transport, food delivery, and blood perfusion in sick arteries in biomedical applications. Figure 7-B clearly distinguishes between peristaltic and retrograde pumping areas by showing that the flow rate rises approximately linearly with the applied pressure difference. Despite the concomitant viscous resistance, higher values of the cilia parameter $\xi$ improve the forward flow for a given pressure rise, suggesting that ciliary motion positively contributes to axial transport. Asymmetric cilia beating creates directional velocity close to the wall, which combines with the peristaltic wave to boost net forward motion. The effective Lorentz force is reduced in the presence of Hall and ion-slip effects, which makes cilia-induced transport processes more important in enhancing volumetric flow within stenosed arteries.

The fact that the pressure rise and friction force curves are oriented inversely with respect to one another indicates that the transition between retrograde and peristaltic pumping regimes is directly related. For $<0$, which is the region of retrograde pumping, the pressure rise is positive and increases (becomes more positive) with the flow rate becoming more negative, which shows that a stronger pressure gradient must be imposed in order to drive the fluid against the natural direction of propagation of the peristaltic wave. At the same time, the friction force stays rather low because of reduced axial momentum. As the critical point ${{Q}}=0$ is approached, the driving peristaltic wave balances the resistive effects due to wall roughness, cilia motion, and stenotic constriction. This represents a purely pumping state with transport of fluid occurring solely by means of the peristaltic mechanism and in the absence of any net imposed flow. Beyond this critical state,${{~Q}}>0$, the system falls into the peristaltic pumping regime characterized by an increase in flow rate, causing the pressure rise to decrease while simultaneously intensifying frictional forces via enhanced velocity gradients arising near the ciliated and rough arterial walls. The point ${{Q}}=0$, therefore, acts as a dynamical threshold separating pressure-dominated and friction-dominated transport mechanisms, highlighting the competing roles of propulsion efficiency and viscous dissipation in stenosed arterial flow.

### Implications for specific factors

The non-dimensional wall shear stress that the fluid applies to the artery wall is represented by skin friction $S{K_f}$. Because increased shear stresses are linked to endothelial damage, plaque rupture, and vascular remodeling, it is an essential biomechanical quantity. The combined impacts of non-Newtonian rheology, wall roughness, and magnetic field effects are also reflected in skin friction in nanofluid-based hemodynamic models.

**Fig. 8:**
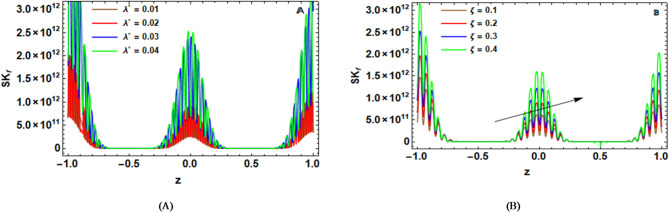
(A) Depicts alternative of $\:{Sk\:}_{f}$ corresponding to various ranges of $\:\:{{\uplambda\:}}^{*}$. (B) Depicts alternative of $\:{Sk\:}_{f}$ corresponding to various ranges of $\:{\upxi\:}$.

Figure 8-A shows how the axial distribution of skin friction along the stenosed artery is affected by the wall roughness pitch ratio ${\lambda ^*}$. The pitch ratio regulates how frequently the flow comes into contact with roughness elements along the wall by representing the spatial frequency of surface imperfections. There is a discernible increase in skin friction when ${\lambda ^*}$ is increased, especially near the stenosed area. This phenomenon can be explained by the fluid-wall interaction being more intense as the distance between roughness peaks increases in its ability to disrupt the near-wall flow. Physically, greater pitch ratios increase wall shear stress by repeatedly upsetting the boundary layer, which amplifies velocity gradients. Thus, the impact of the artery constrictive condition becomes more visible through the increased rate of shear localization. In an applied sense, the increase in skin friction force resulting from alterations in the roughness pitch of the artery walls could be applicable to the atherosclerotic artery situation. In the former condition, uneven depositions of plaques create constricted surfaces.

The impact of $S{K_f}$ is shown in Fig. 8-B with various values of cilia length. The findings show that skin friction along the artery wall is generally improved by longer cilia, with noticeable maxima close to the stenosed section. Longer cilia increase resistance to axial motion and strengthen fluid-structure interaction by physically penetrating deeper into the fluid domain. Stronger momentum exchange and increased shear stresses at the wall are the effects of this. But the cilia also avoid severe localization of stress by redistributing shear forces over a larger area. Understanding mucus transport, regulating microcirculatory flow, and designing bio-inspired microfluidic systems-where cilia-like structures are used to control flow resistance and improve mixing while maintaining manageable shear levels on sensitive surfaces—all benefit from this behavior in real-world applications.

**Fig. 9:**
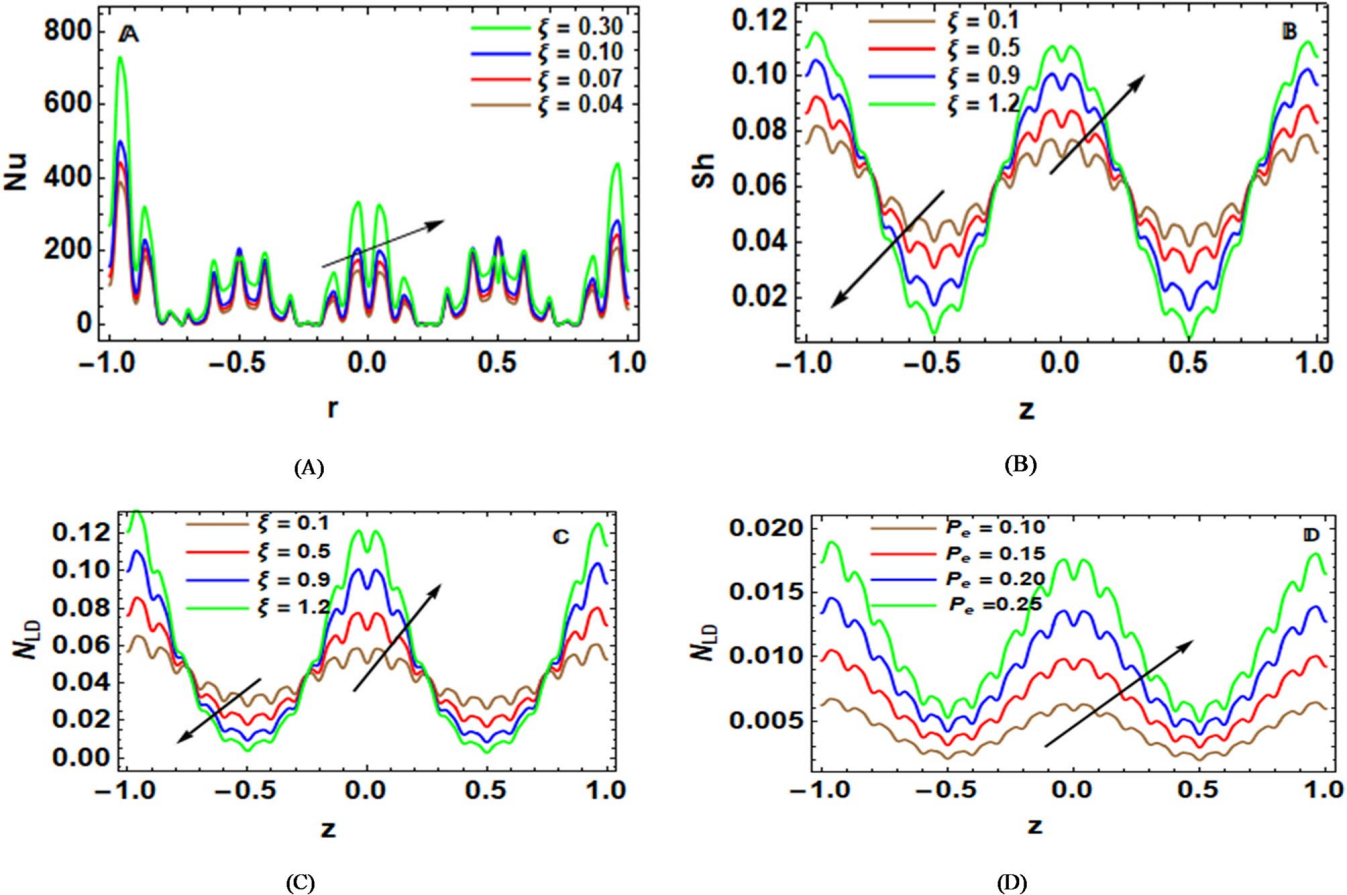
(A) Depicts alternative of $\:Nu$ corresponding to various ranges of $\:{\upxi\:}.\:$ (B) Depicts alternative of $\:Sh$ corresponding to various ranges of $\:{\upxi\:}.\:$ (C) Depicts alternative of $\:{N}_{LD}$ corresponding to various ranges of $\:{\upxi\:}$. (D) Depicts alternative of $\:{N}_{LD}$ corresponding to various ranges of $\:{P\:}_{e}$.

The number given by the Nusselt is a representation of the dimensionless heat transfer rate on the artery walls. Efficient heat transfer within the nanofluid is represented by this numeral. In Fig. 9-A, there is a representation of the effect of $\xi$ on $Nu$. The outcomes show that as $\xi$ is increased, a noticeable enhancement of $Nu$ is found, reflecting a better improvement in convection heat transfer at the wall. From a physical viewpoint, as $\xi$ increases, cilia protrude deeper into the fluid core, which leads to greater disturbances from cilia-induced fluid motions, thus creating a better micro-mixture effect. The fluid motions, in turn, also disturb the thermal boundary layer significantly, thus creating a better transfer of thermal energy from the wall. The increase in wall temperature gradient reflects a better increase in $Nu$; this study has potential applications in various biomedical applications, reflecting a better understanding of ciliated biological conduits in efficient heat transport systems.

Figure 9-B depicts the effect of $\xi$ on the Sherwood factor ${{Sh}}$. It can be noted that ${{Sh}}$ represents the dimensionless rate of the mass transfer on the wall surface. The graph represents the dual effects of the parameter $\xi$ on Sh. It can be noted that in the region$~z \in \left[ { - 1, - 0.2} \right]$, the value of ${{Sh}}$ decreases for an increased value of $\xi$. The increased cilia length creates recirculation zones that lead to the stagnant layers within the area close to the wall surface; the stagnant layersreduce the rate of mass transport by reducing the gradient of the concentrations. In the region $z \in \left[ { - 0.2,1} \right]$, of the z-axis, the increased cilia length enhances the mixing of the cilia and increases the gradient of the concentrations. Therefore, the increased cilia length enhances ${{Sh}}$ by an increased value of the $\xi$.

Figure 9-C demonstrates the effect of ${{\boldsymbol{\upxi}}}$ upon the local density of motile micro-organisms ${{{N}}_{LD}}$. The local density of motile micro-organisms ${{{N}}_{LD}}$ is seen to indicate bio-transport efficiency since it indicates spatial distribution of micro-organisms undergoing a process of active swimming in a fluid. The findings show a significant two-fold effect of $\xi$ upon ${{{N}}_{LD}}$along the axis. The ${{{N}}_{LD}}$values decrease with increasing $\xi$ along the upstream zone $z \in \left[ { - 1, - 0.2} \right]$. A physically intuitive explanation of these trends is as follows: interaction between higher $\xi$ values and local flow disturbances arising through higher cilia length leads to recirculation zones promoting micro-organism dispersion rather than accumulation. Micro-organism transport through increased cilia-induced movements leads to increased scattering and distribution of micro-organisms away from the surface and thus lowers ${{{N}}_{LD}}$. Also, it is observed that increasing values of $\xi$ lead to increased ${{{N}}_{LD}}$values along the downstream zone $z \in \left[ { - 0.2,1} \right]$. A physically intuitive explanation of this trend is as follows: increased coordinated movements through elongated shapes of micro-organisms enhance their micro-mixing and advection towards areas of accumulation. This is a result of velocity gradients favorable to micro-organism transport. This dual behavior arises from the phase-dependent interaction between ciliary motion and the peristaltic wave, where cilia may either disperse or concentrate microorganisms depending on the local flow direction and wall deformation.

Figure 9-D describes how the Péclet factor ${P_e}$ affects ${{{N}}_{{{LD}}}}$. An increase in ${{{N}}_{{{LD}}}}$ results in a marked increase in ${{{N}}_{{{LD}}}}$ within a specified flow domain. Essentially, a high Péclet factor means that advection far exceeds diffusion in spreading micro-organisms in a fluid. As a result, there has to be more advection to help in carrying the non-motile micro-organisms. As a result, there will be more accumulation and less tendency to diffuse. Moreover, with high Péclet factors, there will be more advection in aligning and matching the motion of non-motile micro-organisms with those in a fluid. As a result, there will be more residence time and subsequently a high non-motile density.

### Streamlines and trapping

In this study, the trapping zones or the recirculation regions are taken into consideration as previously formed within the fluid flow through the stenosed capillary. Streamlines of the fluid motion are quite a common means in attempts to visualize and understand the flow behavior in various engineering applications. In the context of the current research, streamlines are taken into account in an endeavor to depict how the pattern of flow would take shape and result in distortion within the stenosed artery due to the combined effects of a number of relevant factors. The factors include, but are not limited to, the ciliated endothelium lining the vessel wall, introducing extra hydrodynamic resistance and giving rise to disturbances in the streamline pattern and thus the trapping zones or recirculation regions. The time-dependent roughness of the vessel wall plays a very significant role in shaping such disturbed flow regions by creating an additional resistance to the flow and hence causing further disruptions in the streamline pattern. The formation of these trapping zones and these disturbed flow regions is of vital significance with regard to certain aspects of fluid dynamics within the system. The flow of vital substances and important cells is affected in certain aspects with regard to these streamlines and phenomena of trapping. Hence, understanding these phenomena of streamlines and trapping is of major significance with regard to improving the dynamics of fluid flow in stenosed arteries. The extensive discussion of ciliated walls and evolving roughness with regard to this MHD-driven artery offers a means of prediction towards advancements in this particular field of study.

**Fig. 10:**
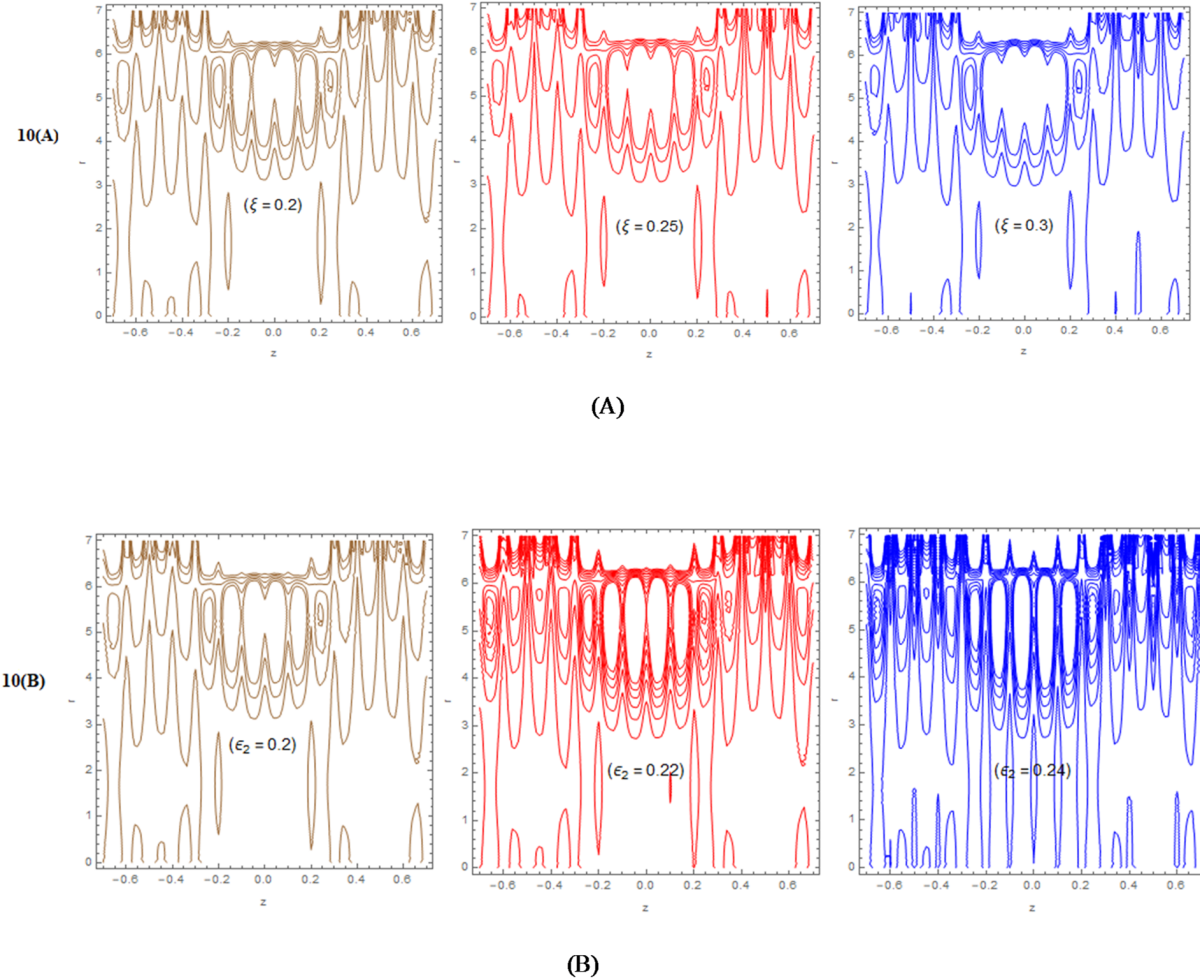
(A) Depicts the streamline patterns corresponding to various ranges of $\:{\upxi\:}.\:$ (B) Depicts the streamline patterns corresponding to various ranges of $\varepsilon _{2}$.

Figure 10-A depicts the streamline configuration for various values of $\xi$. It is obvious again that as the values of $\xi$ increase in the flow field, the streamlines become further distorted. These distortions in the streamlines point to the formation of areas where “trapping occurs in the flow field due to the presence of the ciliated endothelial linings in the capillary. Such cilia in the flow field seem to be almost akin to the hair on the surface of the vessel. These areas of trapping would seem to be accentuated as $\xi$ increases. It would appear physically, in such a trapping situation, the longer cilia would render the flow pattern more complicated. Moreover, the trapping would impede the fluid flowing down the axis. Such trapping could have important ramifications in terms of transporting the more important nutrients, oxygen, etc., through the capillary vessel walls.

Figure 10-B shows how the value of ${\epsilon _2}$ changes the patterns of the streamlines in the fluid flow. It is seen that as the value of ${\epsilon _2}$increases, the distortion in the streamlines, as well as the trapping zones, increase in prominence. The physical reason behind this phenomenon is that the presence of roughness in the walls of the capillary adds to the resistance in the flow, hence creating recirculating zones in the fluid flow. It should be noted that this problem will have significant importance in the context of transporting cells, platelets, etc., in the case of the stenosed capillary.

**Table 1:** Velocity comparison between Sutterby (Ciliated) and Williamson Nanofluid^48^.

Feature / parameter	Our study (Sutterby + Cilia)	Williamson Nanofluid Study (without ciliated wall) [48]
Nanofluid model	Sutterby nanofluid (shear-thinning, more realistic for blood)	Williamson nanofluid (simpler shear-thinning)
Ciliated Endothelium	Present — increases hydrodynamic resistance, slows flow	Absent — only rough wall, no cilia effects
Wall roughness	Dynamic, time-dependent — simulates real arterial deformation	Spatially varying roughness, no cilia interaction
Magnetic Field Effects (Hall & Ion-Slip)	Included, interacts nonlinearly with cilia and wall roughness	Included, interacts with roughness only
Range of Nondimensional Velocity (axial flow)	Approximately **− 1 to ** **+ 1** (very slow, bidirectional due to cilia)	Approximately **0 to ** **163** (fast, unidirectional)
Maximum Velocity Location	Near stenosis peaks and cilia tips, affected by backward flows	Centerline of artery, minimal backward flow
Physical Interpretation	**Small velocities** due to higher viscosity resistance, cilia-induced backflows, and energy dissipation	**High velocities** because simpler fluid model + no cilia + lower internal resistance
Critical Pumping Velocity Behavior	Decreases with increasing roughness amplitude; sensitive to cilia length/eccentricity	Also decreases with roughness amplitude, but absolute velocity remains high

## Validation through experimental and microfluidic studies

A comparison was made of the predictions made with the current theoretical model against previous experimental and microfluidic studies reported in the literature to evaluate the physical realism and credibility of the model. Because of this, there is currently no direct experimental counterpart to one of the intrinsic difficulty To address all features, such as peristaltic motion, rough wall, ciliary activity, and together in the microbe movement, a single experimental setup. Therefore, validation was done by comparing the validity of the dominant physical processes of the model against established experimental data.

### Justification for the selection of experimental studies

Because it directly observes the effect of flow produced by artificial cilia in vitro, the experimental study carried out by Hussong et al.^49^ was adopted as the key study on which the proposed model was based. Key transport phenomena that are the basis of the current model, including cilia-driven advection, deformation, mixing, and recirculation, are directly observed in the study. Additionally, to assess the experimental consistencies of cilia-induced transport properties with various platforms of microfluidics devices, the detailed study provided by Sahadevan et al.^50^ was considered as an assistant study to assess the practicality of cilia influences provided by the current theory by considering this as a review paper that contains some experimental properties supporting the physical practicum of cilia influences considered by this study.

### Physical correspondence between the present model and experimental studies

The micro-scale transport mechanisms, such as ciliary movements, are the concern of the existing model, as well as that adopted for the experimental study. The synthetic cilia, by employing a magnetic actuation method, display complex streamlining, enhanced advection, and directionality, a phenomenon also witnessed under the existing model owing to cilia thickness, peristaltic waves, as well as utilizing the geometry. The effects of deformation of the walls and the nature of the surface roughness included within the current simulation support the experiments identifying the significant impact of such characteristics on the velocity fields and transport efficiencies to an even greater degree. The primary processes for the generation of the flow are physically consistent without the inclusion of the peristaltic motion within the experimental scenarios.

### Comparative analysis

**Table 2:** Comparison between experimental observations and present theoretical predictions.

Feature	Hussong et al. (2011) – experimental^49^	Present theoretical model	Agreement
Flow generation mechanism	Artificial cilia actuation	Cilia motion + peristaltic wave	Consistent
Flow regime	Low Reynolds number microflow	Low Reynolds number assumption	Consistent
Velocity behavior	Enhanced velocity near cilia tips	Velocity amplification near ciliated walls	Strong agreement
Streamline structure	Curved and distorted streamlines	Closed and distorted streamlines	Strong agreement
Recirculation / trapping	Localized vortices observed	Trapped bolus and recirculation zones	Qualitative agreement
Transport enhancement	Increased advection dominates diffusion	Increased Peclet-driven transport	Strong agreement
Wall geometry influence	Microchannel confinement	Roughness and stenosis effects	Complementary

### Discussion of validation results

The aforementioned comparison reveals that the predicted trends based on current theory, in addition to the experimental trends, are in good qualitative agreement with each other. It was established through both methods that the motion of the cilia has a key influence on the improvement in the advection processes, in the topology of the streamlines within the flow region, and the establishment of recirculating zones within the flow region. In addition, the predicted improvement in the flow processes of heat transfer, mass transfer, and microbes according to the current theory was confirmed through the experimental evidence of the improvement in the velocity of the flow or distortion in the streamlines of an artificial system of cilia present in the flow region. Even though non-Newtonian rheology and peristaltic motion on the wall have not been included in the experiment, the basic consistency between cilia-induced transport routes supports the physics on which the current model is based. On one hand, the addition of peristalsis, roughness, and non-Newtonian effects to the theoretical model extends the applicability of physical principles presented in the experiment to more physiologically valid processes. Then, all aspects considered, the current model is effective and physically valid in portraying major microfluidic transport routes and extends experimentally supported physical processes to complex peristaltic and biological flows.

The analogy with the results of experimental investigations, on the other hand, is of physical significance regardless of the dimensionless character of the current study, since due to the possibility to distinguish the governing processes and specific geometric scales and conditions by resorting to dimensionless variables, the use of the results of experimental investigations may be found frequently within the scope of the study of the transport phenomena of fluids. The trends of the results characterizing the superior enhancement of the transport rate due to the motion of the cilia, as well as the deformation of the streamlines and the enhancement of the transport rate, depend on the specific study of the trends of the results rather than the specific numerical quantities of the results of the problem under consideration, regardless of the intrinsically dimensional character of the results of the problem obtained according to the results of the investigations published within the corresponding literature.

## Conclusion framework

The study aimed to explore the combined impacts of the processes that involve a chemical reaction, exposure to radiation, heat transfer, and strong magnetization. Also, its impact on the flow of the Sutterby nanofluid was evaluated as it passed through a stenosed arterial tube that was immersed in the medium and was lined by ciliated material. The model sought to assess the active involvement of cilia to improve the contact of the fluids with the surface as a way to regulate the characteristics of flow. All the processes that influence flow, such as the Joule heat, constriction caused by stenosis, Hall and ion slip reactions, Darcy-Forchéimer flow obstructions, etc., are known to affect the fluid flow and its properties. To enhance the actual physical reliability of the design, a developed Arrhenius theory is joined with the Buongiorno theory of the nanofluid computer simulation. The equations that direct the phenomena are smoothed utilizing the long wavelengths theory and low esteem of the Reynolds volume calculations, while the solutions from the scrutinizing analytical investigations are found through a homotopy perturbation technique, whereby a wide-ranging parametric design has been found through the computer simulations used in the current investigations in the following domains: velocity, temperature, concentration, pressure increase, friction force, density of microbes. An insightful discussion of critical transportation properties, e.g., skin friction, Nusselt amount, Sherwood quantity, along with local density of the migrating microbes, demonstrates the coupled impacts of cilia, magnetic, roughness, as well as non-Newtonian effects during peristaltic biofluid transportation.

The following points succinctly indicate the major findings of this study:

1. By enhancing the mechanical interaction between the fluid and the moving wall, longer cilia in a peristaltic artery significantly alter the structure of near-wall fluid flow. Because the cilia are longer, these cilia extend deeper into the fluid-flow stream, increasing drag on the fluid. Consequently, the speed of the fluid is reduced significantly.
2. In the face of existing roughness and stenosis, an increased level of cilia eccentricity invariably leads to a recognizable rise in $\:w\left(r\right).$.
3. In spite of cilia-generated motion and moderate levels of stenosis, $\:w\left(r\right)$ decreases as the amplitude ratio for roughness in the walls increases.
4. When cilia motion and minor stenosis occur, an increase in the pitch of the wall roughness causes an increase in the velocity.
5. The nonsymmetrical surface from the combination of cilia and surface roughness increases the shear events, changes the flow locally, and creates micro-vortices along the vessel surface. Also, from the image, one notices the presence of positive and negative $\:{H}_{1}\:(z;t)\:$for the forward and backward motion of the cilia over the whole cycle of the peristalsis.
6. With an increase in the Sutterby parameter, the shear-thinning characteristics of the fluid increase, hence its apparent viscosity at low shear rates. Its consequence is a marked fall in axial velocity in small artery segments due to increased flow resistance.
7. Fluid temperature increases with the increase in roughness amplitude and the eccentricity of the cilia’s elliptical beating pattern.
8. As $\:{{{\rm\:E}}}_{a}$ increases, the concentration across the flow decreases due to the reduction in mass transfer rate.
9. A larger $\:{P}_{e}$ implies that, under conditions where advection is stronger than diffusion, microorganism transport in the direction of flow will be enhanced.
10. There is a linear relation between pressure rise and flow rate, with ciliary activity increasing flow resistance and reducing pumping efficiency, especially in stenosed arteries. Nevertheless, higher cilia intensity enhances forward transport at a given pressure rise due to its constructive interaction with the peristaltic wave.
11. Increasing the wall roughness pitch ratio intensifies skin friction, particularly near the stenosis, due to stronger boundary-layer disruption and enhanced wall shear stress. In addition, longer cilia increase skin friction along the arterial wall, with peaks near the stenosed region, as deeper cilia penetration strengthens fluid–structure interaction and wall shear.
12. An increase in the normalized cilia length enhances the Nusselt number, indicating improved convective heat transfer due to cilia-induced mixing and thermal boundary-layer disruption.
13. The cilia length exhibits a dual effect on the Sherwood number, reducing mass transfer upstream due to recirculation while enhancing it downstream through intensified mixing.
14. The normalized cilia length causes a dual response in microorganism density, decreasing upstream due to dispersion and increasing downstream due to enhanced directed transport and accumulation.
15. Increasing the cilia length intensifies streamline distortion and trapping zones due to enhanced cilia–fluid interaction, leading to stronger recirculation and reduced axial transport. Also, higher wall roughness amplitude​ amplifies streamline deformation and trapping regions by increasing flow resistance, highlighting the combined role of roughness and cilia in modifying hemodynamics within stenosed capillaries.
16. The comparison between this model and the experimental study by Hussong et al.^49^ shows strong qualitative agreement between the present model and experiments, confirming the dominant role of ciliary motion in enhancing advection, streamline distortion, and transport processes. Despite using dimensionless variables, the model successfully captures scale-independent transport trends consistent with experimental observations of cilia-induced flow enhancement.

### Practical implications

**Important** insights into microfluidic transport processes are provided by this investigation of the peristaltic flow of a Sutterby nanofluid over ciliated and rough surfaces that contain motile gyrotactic bacteria. Heat, mass, and microbe axial transfer, recirculation zones, and fluid velocity are all significantly impacted by ciliary action and wall roughness. The results have immediate practical implications, such as:

- Drug delivery with specificity in ciliated biological channels.
- Transport of nutrients and oxygen in microcirculatory systems.
- Motile microorganism manipulation and control for biological applications.
- Design of bioinspired microfluidic devices for effective mass, heat, and mixing.
- Lab-on-a-chip devices for medication administration and diagnosis.
- Filtration and waste elimination in microfluidic systems.
- Heat exchanger and energy transfer optimization in micro-scale flows.
- Creation of artificial cilia systems to enhance flow control in engineering and healthcare environments.

Furthermore, the results of the peristaltic flow analysis of Sutterby nanofluid can be applied to targeted drug delivery systems that utilize enhanced nanoparticle transport, optimization of microfluidic devices for precise fluid and particle control, and the design of advanced stents and vascular grafts that account for wall roughness and complex hemodynamics. Further applications include biomedical heat and mass transfer systems for improved thermal management of biological fluids, lab-on-a-chip platforms for studying microorganism behavior and bioconvection effects, and optimization of biosensors relying on ion transport and electrokinetic phenomena. These practical implications underscore the real-world relevance of the proposed model.

Moreover, the stenosed artery with ciliated endothelium and wall roughness can be considered as a representative domain for engineering processes involving peristaltic transport in deformable channels. Similar mechanisms occur in microfluidic pumps, biomedical devices, and heat exchangers with structured surfaces, where surface motion and roughness influence flow, mixing, and transport. Modeling the domain in this way provides insights that are applicable beyond physiology, guiding the design and optimization of devices that rely on controlled fluid motion in confined or deformable conduits.

### Limitations

As far as the modeling technique used in this present paper is concerned, a few of its limitations can be identified:

1. Simplified Artery Geometry: Although the research seeks to investigate the flow of fluids in human arteries as part of the human circulatory system. The investigations would be unable to account for the complexity observed in the real system because of the dependency on idealized arterial geometries with a simplified description of the stenosis, the surface roughness, and cilia.
2. Properties of nanofluids and behaviors of microorganisms are studied under consistent circumstances.
3. The study mainly refers to the assumption of a low Reynolds number and extended wavelength.
4. High model complexity due to multiple coupled phenomena.
5. Idealized assumptions (low Reynolds number, long wavelength, simplified artery geometry).
6. Limited quantitative validation; current comparisons are mainly qualitative.

### Future aspects

Motile gyrotactic organism transport, entropy production, ion flow, enhanced Darcy–Forchheimer flows, and peristalsis of non-Newtonian fluids, such as Casson and Carreau fluids, will incorporate ciliated surfaces, couple stresses, and micropolar fluid features. Also, simplify the model and perform sensitivity analysis for key parameters. Quantitative experimental validation using in vitro or clinical data. Explore computational approaches (e.g., CFD) for biomedical applications. It has thus presented a clear understanding of various fluid transport mechanisms with respect to non-Newtonian fluids and biological tissues. Moreover, these areas would enable researchers to create complex simulation and prediction models, and would thus find valuable applications in designing various biological and non-biological devices.

## Data Availability

The data supporting the findings of this study are fully provided within the manuscript.

## List of symbols

$a$: The disseminating peristaltic movement’s waveform amplitude (m)
$A_{1}$: The medium’s shear deformation (s^− 1^)
$\underline {B}$: Magnetic field intensity applied externally (T)
$B_{0}$: Homogeneous magnetic field strength (T)
$B_{r}$: Brickman numeral (dimensionless)
$b$: Microorganisms’ chemotactic coefficient (m/s)
$b_{1}$: Height of wall roughness (m)
$$C$$: Concentration (kg/m^3^)
$C_{1}^{*}$: Concentration of arteries (kg/m^3^)
$$C_{b}$$: Forchheimer coefficient (m^2^/s)
$C_{e}$: Nanoparticle factor (dimensionless)
$C_{f}$: Specific temperature of the medium (J/(kg·K))
$C_{p}$: Small particle specific temperature (J/(kg·K))
$C_{s}$: Susceptibility to concentration (dimensionless)
$D$: Component of mass diffusivity (m^2^/s)
$D_{B}$: Brownian diffusion factor (m^2^/s)
$D_{Ma}$: Modified Darcy amount (dimensionless)
$D_{m0}$: The diffusivity of the microorganisms (m^2^/s)
$D_{T}$: A nanoparticle’s thermophoretic diffusion coeffecient (m/K)
$D_{u}$: Dufour parameter (dimensionless)
$$\widehat{{\underline {\mathrm{E}}}}$$: Electric field (V/m)
$$E_{a}$$: Activation energy (J)
$E_{c}$: Eckert coefficient (dimensionless)
$F_{r}$: Forchheimer numeral (dimensionless)
$G_{r}$: Thermal Rayleigh parameter or thermal Grashof factor (dimensionless)
$$\underline {g}$$: Gravity (N)
$H^{2}$: Hartmann coefficient (dimensionless)
$$H_{0}$$: Stenosis height (m)
$H_{1}$: Surface area of the wall (m)
$h$: The greatest extent of stenosis (m)
$I$: Matrix of identity (dimensionless)
$\underline {J}$: Density of current from electricity (A/ m^2^)
$K$: Conductivity of heat (W/(m·K))
$K_{0}$: The Rosseland mean absorption factor (m^2^/kg)
$K_{r}$: Reaction reagent (mol/( m^3^·s))
$K_{T}$: Thermal diffusion component (dimensionless)
$$k$$: Thermometric conductivity (W/(m·K))
${\mathrm{k}}^{*}$: Moderate porous permeability (m^2^)
$$\frac{k}{{R_{1} }}$$: Speed (m/s)
${\mathrm{m}}$: Power-law exponent or flow behavior index (dimensionless)
$m_{1}$: Slope of the exterior tube (dimensionless)
$$N$$: Microorganism concentration (1/ m^3^)
$N_{1}^{*}$: Microorganism that moves at the pipe (1/ m^3^)
$N_{b}$: Brown’s movement coefficient (without dimensions)
${\mathrm{N}}_{LD}$: The local density of microorganisms with mobility (
$N_{t}$: Thermophoresis coefficient (dimensionless)
${\mathrm{Nu}}$: Nusselt coefficient (dimensionless)
$n_{1}$: Material time constant (s)
$n$: Fitted frequency (dimensionless)
$$P$$: Pressure (N/m^2^)
$P_{e}$: Peclet coefficient (dimensionless)
$P_{r}$: Prandtl coefficient (dimensionless)
$q_{r}$: Radiative inflow-Kelvin (K)
$Q_{0}$: Heating by friction (W/m^2^)
$Q^{*}$: Frictional heating without dimensions
$R\left( z \right)$: The tapered pipe’s radius (m)
$R_{1}$: The constant pipe’s radius (m)
$R_{b}$: Biconvection Rayleigh coefficient (dimensionless)
$R_{D}$: Darcy resistance (dimensionless)
$R_{N}$: Particles of nanoparticles Grashof numeral (dimensionless)
$R_{n}$: Radiation coefficient (dimensionless)
$S_{c}$: Schmidt coefficient (dimensionless)
${\mathrm{S}}_{Tb}$: Sutterby substance (dimensionless)
${\mathrm{Sh}}$: Sherwood coefficient (dimensionless)
$SK_{f}$: The friction of the skin (dimensionless)
$$T$$: Temperature (K)
$T_{e}$: Expansion of thermal volume (1/K)
$$T_{m}$$: Average temperature (K)
${\mathrm{T}}_{1}^{*}$: The pipe’s temperature (K)
$\underline {\mathrm{V}}$: The fluid’s speed (m/s)
$z_{0}$: The halfway point of stenosis (m)
${\updelta }$: Proportionality of the length of wave to a dimensionless distinctive length (dimensionless)
$\phi$: Structure’s tapering angle (dimensionless)
${\Phi }$: Viscous Dissipation (kg m^−1^ s^− 3^)
$\pi$: The second invariant according with strain tensor (s^− 2^)
${\upmu }_{f}$: Viscosity under lower yield stress (kg/m s)
${\upxi }$: The normalized measure of cilia length relative to the characteristic scale (dimensionless)
${\upxi }_{0}$: The undisturbed reference configuration of the cilia wall (m)
${\upalpha }$: Measure of the eccentricity of the elliptical beating pattern of cilia (dimensionless)
${\upzeta }\left( {{\mathrm{Z}},{\mathrm{t}}} \right)$: Horizontal displacement of the cilia tips
${\upeta }\left( {{\mathrm{Z}},{\mathrm{t}}} \right)$: Radial displacement of the cilia tips
$$\lambda$$: Wavelength (m)
$$\lambda_{1}$$: Roughness pitch (m)
$\lambda^{*}$: Roughness of walls pitch proportionality (m)
$\rho$: Total density (kg/m^3^)
$\rho_{f}$: The density of the liquid (kg/m^3^)
$\rho_{m}$: The microorganism density (kg/m^3^)
$\rho_{p}$: The nanoparticle density (kg/m^3^)
$(\rho c)_{f}$: Fluid’s heat capacity (J/(kg·K))
$(\rho c)_{p}$: Nanoparticle’s effective heat capacity (J/(kg·K))
$\sigma_{hnf}$: The nanofluid’s effective electrical conductance (S/m)
$\sigma_{0}$: Stefan Boltzman’s constant (W/(m^2^·K^4^))
$${\upsigma }^{*}$$: Stress tensor(N/m^2^)
$\vartheta$: Microorganisms’ average volume (m^3^)
$$\omega^{*}$$: Boltzmann consistency (J/K)
$\omega_{c}$: The speed at which a cell can swim (m/s)
$\varpi_{e}$: The frequency of cyclotrons (Hz)
$\varepsilon _{1}$: Percentage of the wave’s amplitude (dimensionless)
$\varepsilon_{2}$: Percentage of the amplitude of wall roughness (dimensionless)
$$\varepsilon^{*}$$: Porosity (dimensionless)
$\hat{\alpha }$: Constant rate of reaction (dimensionless)
$\alpha_{e}$: Ion slip factor (dimensionless)
$\beta$: Adverse temperature gradient (dimensionless)
$\beta_{e}$: Hall amount (dimensionless)
$\beta_{i}$: Ion-slip amount (m/s)
$\beta_{t}$: Temperature ratio (dimensionless)
$$\gamma$$: Slip coefficient (m/s)
$\gamma^{*}$: Dimensionless slip coefficient
$\dot{\gamma }$: Rate-of-strain tensor (s^− 1^)
$\user2{\rm E}$: Energy of activation component that is not dimensional
$$\tau$$: Shear stress (N/m^2^)
$\tau_{e}$: Time of electron collision (s)
${\Xi }$: The rate of fluid volume transport in a fixed geometry
$\hat{Q}$: Averaged over time circulating rate
